# Membrane-permeable trehalose 6-phosphate precursor spray increases wheat yields in field trials

**DOI:** 10.1038/s41587-025-02611-1

**Published:** 2025-04-29

**Authors:** Cara A. Griffiths, Xiaochao Xue, Javier A. Miret, Fernando Salvagiotti, Liana G. Acevedo-Siaca, Jacinta Gimeno, Matthew P. Reynolds, Kirsty L. Hassall, Kirstie Halsey, Swati Puranik, Maria Oszvald, Smita Kurup, Benjamin G. Davis, Matthew J. Paul

**Affiliations:** 1 Rothamsted Research, Harpenden, UK; 2 Department of Chemistry, University of Oxford, Oxford, UK; 3 Crops, Soil and Water Management Group, Agronomy Department, EEA Oliveros INTA, Santa Fe, Argentina; 4 National Council of Scientific and Technical Research (CONICET), Buenos Aires, Argentina; 5 Global Wheat Program, International Maize and Wheat Improvement Centre (CIMMYT), Texcoco, Mexico; 6 Department of Pharmacology, University of Oxford, Oxford, UK; 7 Rosalind Franklin Institute, Harwell, UK; 8 Present Address: Horticulture and Product Physiology, Wageningen University, Wageningen, The Netherlands; 9 Present Address: Department of Statistics, University of Warwick, Coventry, UK

**Keywords:** Field trials, Glycobiology, Plant signalling, Drought

## Abstract

Trehalose 6-phosphate (T6P) is an endogenous sugar signal in plants that promotes growth, yet it cannot be introduced directly into crops or fully genetically controlled. Here we show that wheat yields were improved using a timed microdose of a plant-permeable, sunlight-activated T6P signaling precursor, DMNB-T6P, under a variety of agricultural conditions. Under both well-watered and water-stressed conditions over 4 years, DMNB-T6P stimulated yield of three elite varieties. Yield increases were an order of magnitude larger than average annual genetic gains of breeding programs and occurred without additional water or fertilizer. Mechanistic analyses reveal that these benefits arise from increased CO_2_ fixation and linear electron flow (‘source’) as well as from increased starchy endosperm volume, enhanced grain sieve tube development and upregulation of genes for starch, amino acid and protein synthesis (‘sink’). These data demonstrate a step-change, scalable technology with net benefit to the environment that could provide sustainable yield improvements of diverse staple cereal crops.

## Abstract (web summary)

A sunlight-activated chemical spray increases wheat yield in field trials across rainfall conditions.

## Main

Global challenges to food security are greater now than at any time in the modern era^1^. Cereals supply most of the world’s food^2^. As one of the major staple food sources around the world, wheat (*Triticum aestivum* L.) provides approximately 20% of the calories and protein to the daily human diet^3^. Steady breeding progress has increased potential wheat yield globally at about 0.6% per year^4^. Actual wheat production depends on the area cultivated and its realization of yield potential. From 2018 to 2022 (ref. ^5^), global wheat production increased from 732 million tons to 770 million tons (+0.49% per year). Global population is expected to grow by a billion by 2050 to 9 billion^5^. In the Global South (https://unctadstat.unctad.org/EN/Classifications.html), more than 1.5 billion resource-poor people depend on a constant and affordable supply of wheat as their main staple^6^.

The Green Revolution of the 1960s that saw much-needed increases in crop yield has since required more fertilizer^7^. However, for any new technology to have a large impact on agricultural production, yield increases will need to be achieved sustainably^8^, ideally without extra fertilizer input. Fertilizer manufacture generates CO_2_ and increases costs for farmers and consumers, and use generates emissions of the potent greenhouse gas N_2_O from soils. Furthermore, fertilizer runoff degrades water sources and aquatic ecosystems^9^. The variability of rainfall is predicted to increase under climate change^10^, and, therefore, a further critical challenge is to increase crop yield under altered weather patterns and, in particular, variable rainfall^11^. For meaningful impacts on food security, increased yield potential must necessarily be combined with resilience to suboptimal water availability.

Yield is a polygenic trait; it is difficult to determine the most effective genes that result in high-yielding progeny in crops. Molecular breeding, genetic modification and gene editing approaches offer promise through both genetic range and precision, but they take time to deliver breakthroughs for yield and may not be accepted in all regions^12–16^. Many targeted yield improvements in laboratory and controlled environments that show promise do not deliver yield improvements in the field^17^, creating a major obstacle to harnessing genetic and other technologies for food security. New transformative technologies are required.

The major physiological determinants of yield and yield components in wheat are before anthesis in the formation of reproductive tillers and the formation of spikelets and florets within spikelets in the developing spikes of these tillers. These traits are determined by both developmental genes and photosynthetic activity and are regarded as being ‘source limited’^18^. Fertilization of florets at anthesis (fruiting efficiency) affects potential grain numbers at harvest^19^. After anthesis, grain filling and grain retention are the major physiological determinants of yield. In contrast to physiological events before anthesis, it is considered that wheat yield is ‘sink limited’ during grain filling^18^. Sink strength during grain filling is determined largely by metabolic flow of carbon into starch, which could be targeted for yield improvement. However, there are no examples of successful specific targeting of starch biosynthesis that increase yield in wheat or other crop varieties.

Trehalose 6-phosphate (T6P) is a major plant sugar signal and metabolic regulator that inhibits the protein kinase SnRK1 (refs. ^20,21^). SnRK1 is a member of the conserved AMPK/SNF1 protein kinase master regulators of metabolism, sugar and energy homeostasis found in all organisms^22^. In plants, inhibition of SnRK1 by T6P promotes anabolic pathways, including starch biosynthesis^20,23,24^, making T6P an attractive target for yield improvement, yet very little is known about the impact of T6P on the starch pathway in harvested crop sinks^24^. Previous work showed that genetic modification of the T6P pathway improved yield in single varieties in field conditions in maize^25^ and rice^26^; however, this has been difficult to show in wheat.

**Fig. 1:**
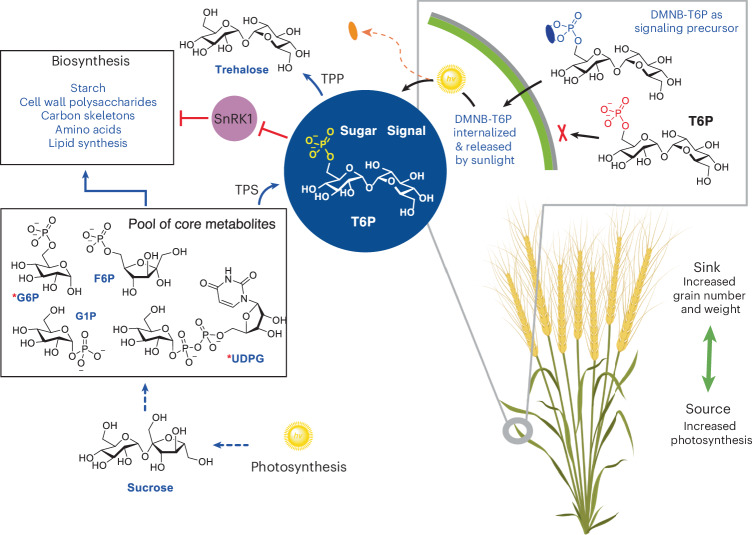
Summary of the ‘signaling precursor’ strategy for modulation of the T6P pathway. Use of a plant-permeable precursor of T6P, DMNB-T6P, that is released by sunlight allows dose-varied control of the T6P pathway. Parts of this figure were created with BioRender, Davis, B. (2025): https://BioRender.com/p09q308.

To alter T6P levels in wheat without need for genetic methods and breeding, we previously developed a method for chemical intervention using plant-permeable analogs of T6P designed and constructed based on a signaling precursor concept (Fig. 1) for permeability, ready uptake and sunlight-triggered release of T6P in planta^23^. A chemical spray application under controlled conditions suggested possible benefits over genetic methods and breeding, including flexible timing and modulated release of T6P. Such direct chemical technology enables small adjustments (for example, dosing) to potentiate plant function across a variety of crops rather than complete genetic engineering of each new crop or variety and/or for each specific growing region. Such a biostimulant based on T6P has the potential for widespread applicability and the possibility to extend physiological limits simply through a well-timed pulse of T6P^23^. In a previously published controlled environment, yield of spring wheat Cadenza was increased up to 18% through increasing grain weight and starch content when T6P spray was applied during the grain filling period^23^. However, to be of value in agriculture, such effects would need to be demonstrated in wheat varieties grown under typical, variable, field conditions—increasing starch biosynthesis while also alleviating sink limitation during grain filling to increase yields under periods of drought, without requirements for increased nitrogen fertilizer or irrigation inputs.

Here we demonstrate a trial using T6P precursor spray into field wheat production over 4 years in an agricultural system with yields close to the global average of 3.6 tons per hectare^5^, with variable patterns of high and low rainfall. These field trials, spraying three elite bread-making varieties, coupled with a field trial testing four additional varieties in an optimal, high-radiation, irrigated spring wheat environment, show yield increases that provide a much-needed step change for wheat production. Mechanistic analyses reveal upregulation of the pathway from sucrose to starch in grain, upregulation of genes for amino acid and protein synthesis and increased flag leaf photosynthesis that drive an elevation of both ‘source’ and ‘sink’, timed during early grain filling. Controlled environment trials suggest that future expansion to additional crops will be possible.

## Results

### Ready synthesis of T6P precursor enables large-scale field trials

**Extended Data Fig. 1:**
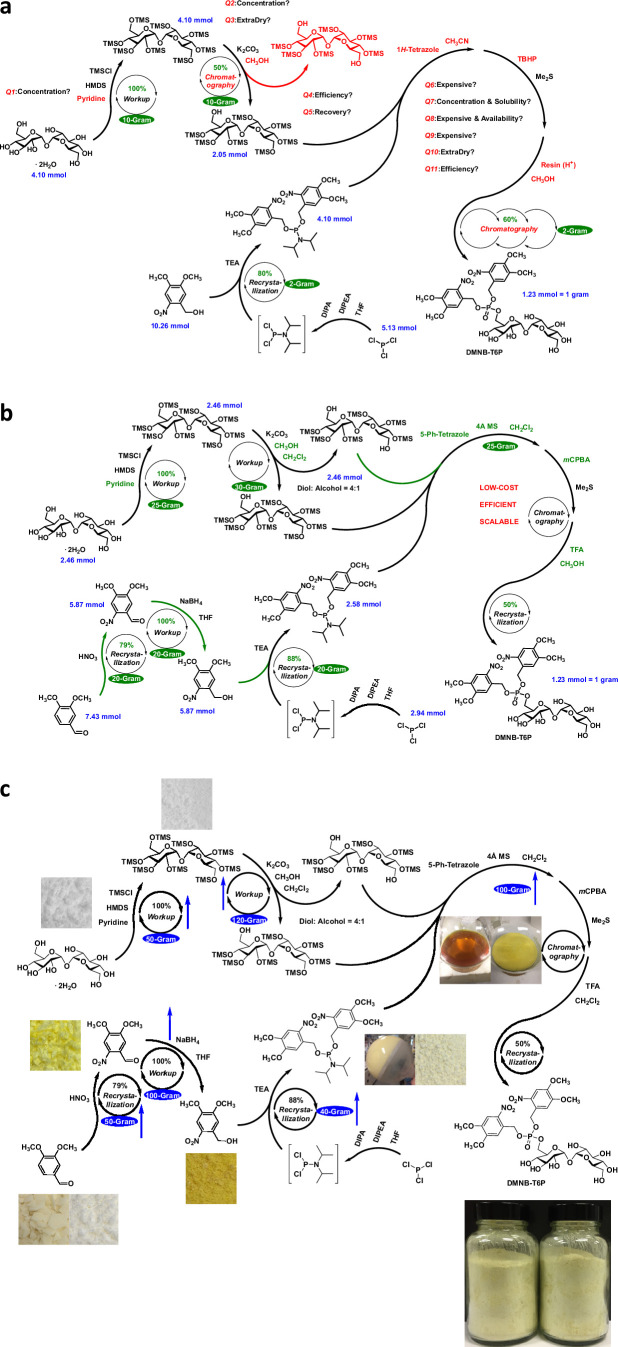
Summary of the development of a distributable low-cost, scaleable synthetic method for DMNB-T6P that enabled field-scale trials. **(a)** Initial synthetic route. **(b)** Improved route based on optimization of key steps. **(c)** Adaptation of route to crystallization of key intermediates and final product DMNB-T6P (bottom right).

The wide-scale adoption of signaling precursor chemical technology necessitates distribution of the associated chemical synthetic methods for its larger-scale synthesis. This, in turn, requires a method that would transform prior syntheses of the sunlight-activated T6P precursor DMNB-T6P from sub-gram quantities, sufficient only for small-scale, controlled-environment experiments^23^, to industrializable 100-gram (g) to kilo-scale methodology for field trials and global farming. Previous synthetic routes used chromatography and other purification processes and steps that were inefficient and difficult to scale^23^. Effective chemical synthetic access was, therefore, achieved via a redesigned route that allowed crystallization of vital intermediates, as well as the final target compound, DMNB-T6P, as solids (Extended Data Fig. 1). This crystallization technology is scalable and distributable. The accessibility of the newly developed route for non-specialist industrialization was confirmed by independent verification at three contract research organizations, allowing routine generation of >50-g batches sufficient for international distribution and application in global field trials. Purified powder is stable in the dark at room temperature for at least 2 years.

### DMNB-T6P microdose application increases yield in elite wheat

**Extended Data Fig. 2:**
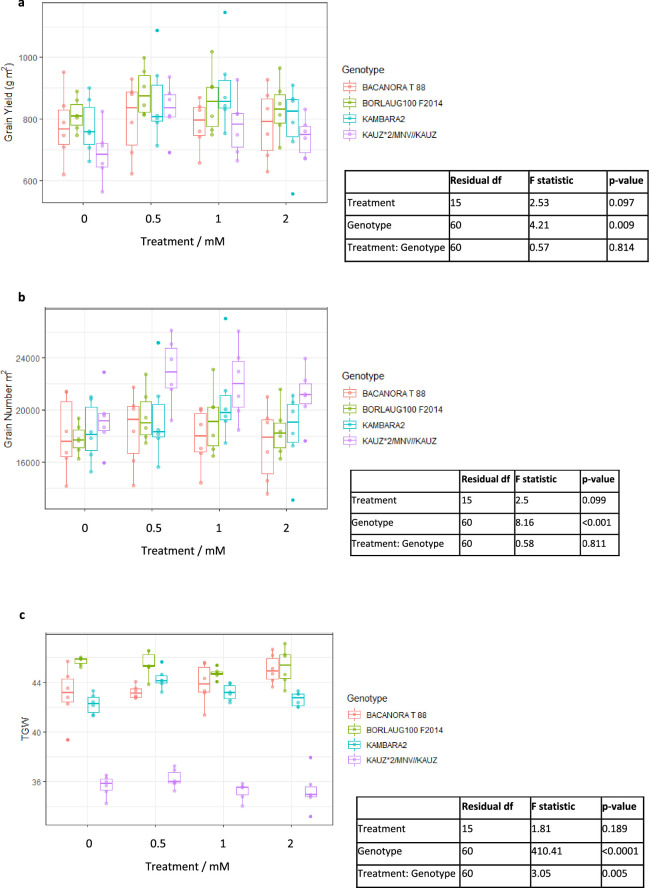
Data from scoping trial in Mexico. **(a)** Boxplot distribution for grain yield. Four genotypes BACANORA T 88, BORLAUG100 F2014, KAMBARA2, KAUZ*2/MNV/KAUZ were treated with three concentrations of DMNB-T6P (0.5, 1, and 2 mM DMNB-T6P compared to control 0 DMNB-T6P) at the experimental field station Campo Experimental Norman E. Borlaug (CENEB) in Ciudad Obregon, Sonora, Mexico. Each dosage contains 24 observations (n = 24). **(b)** Boxplot distribution for grain number per m^2^. Four genotypes BACANORA T 88, BORLAUG100 F2014, KAMBARA2, KAUZ*2/MNV/KAUZ were treated with three concentrations of DMNB-T6P (0.5, 1, and 2 mM DMNB-T6P compared to control 0 DMNB-T6P) at the experimental field station Campo Experimental Norman E. Borlaug (CENEB) in Ciudad Obregon, Sonora, Mexico. Each dosage contains 24 observations (n = 24). **(c)** Boxplot distribution for thousand grain weight (TGW). Four genotypes BACANORA T 88, BORLAUG100 F2014, KAMBARA2, KAUZ*2/MNV/KAUZ were grown at three concentrations of DMNB-T6P (0.5, 1, and 2 mM DMNB-T6P compared to control 0) at the experimental field station Campo Experimental Norman E. Borlaug (CENEB) in Ciudad Obregon, Sonora, Mexico. Each dosage contains 24 observations (n = 24). 5 degrees of freedom analysis was conducted using multi-strata ANOVA to account for the split-plot design. Models were fitted in Genstat 22nd edition. Box plots range from the first (Q1) to the third quartile (Q3) of the distribution and contain the 25th to 75th percentiles of dataset, respectively, representing the interquartile range (IQR). The centre line inside each box represents the median value (50th percentile). Whiskers extending below Q1 and above Q3 denote minimum and maximum values of the dataset within 1.5 x IQR from the 25th and 75th percentiles, respectively. Each value beyond these upper and lower bounds are outliers represented by dots above or below the whiskers.

We performed a scoping field trial under standard irrigated conditions at the Global Wheat Program, International Maize and Wheat Improvement Center (CIMMYT), in Obregon, Mexico, a site that has been used in the selection of higher yielding wheat for distribution particularly to the Global South regions. One volume (a dose 2 equivalent) was used (Supplementary Table 1) at three concentrations (0.5 mM, 1 mM and 2 mM) of the signaling precursor DMNB-T6P sprayed at 10 days after anthesis (DAA). Yield data (+9–22% increase, average 15.3% at 0.5 mM and 1 mM DMNB-T6P) were observed in four varieties (*P* = 0.097; Extended Data Fig. 2a) of sufficient promise to justify larger-scale trials.

To test full robustness under the major environmental constraint limiting food security—that is, variable rainfall^11^—field trials were conducted over four seasons (2018, 2020, 2021 and 2022) at the National Institute of Agricultural Research (INTA) Oliveros Research Station (Santa Fe, Argentina). DMNB-T6P was applied once at 1 mM in two or three volumes (dose sizes) in each year (dose sizes 1, 2 or 3 = 220 ml, 438 ml or 656 ml, respectively, per 7-m^2^ plot, equivalent to 255 g per hectare, 510 g per hectare and 765 g per hectare; Supplementary Table 1). Application was again at 10 DAA in 2018, 2020 and 2022 and at 16 DAA in 2021 (delayed due to late shipping of DMNB-T6P during the coronavirus disease 2019 (COVID-19) pandemic; Supplementary Table 2).

**Fig. 2:**
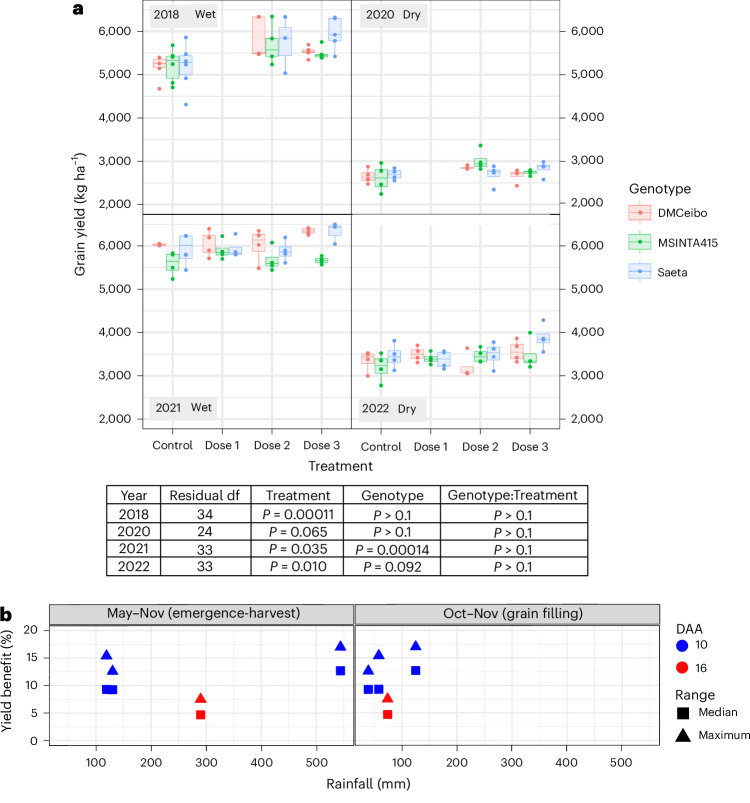
Wheat grain yield (kg per hectare) in response to DMNB-T6P spray over four seasons. **a**, Three spring wheat varieties, DM Ceibo, MS INTA 415 and Saeta, grown 2018–2022 in wet and dry years at two dose rates of DMNB-T6P in 2018, 2020 and three dose rates in 2021, 2022 compared to control with no DMNB-T6P (*P* = 0.00011, *P* = 0.065, *P* = 0.0348 and *P* = 0.010 for 2018, 2020, 2021 and 2022, respectively). Each data point represents an individual field plot (*n* = 5 in 2018; *n* = 4 in 2020, 2021 and 2022). Statistical analysis of each Argentinian field trial was performed using a two-way factorial ANOVA accounting for the randomized complete block layout in R version 4.2.1. Additionally, a combined analysis over all 4 years was performed using a mixed model framework fitted using REML (Supplementary Table 3). All data are shown on harmonized scales; for plots with expanded scales, plots with means and comparison of SEDs of combined means and individual means, see also Extended Data Fig. 10. Box plots range from the first quartile (Q1) to the third quartile (Q3) of the distribution and contain the 25th to 75th percentiles of the dataset, respectively, representing the interquartile range (IQR). The center line inside each box represents the median value (50th percentile). Whiskers extending below Q1 and above Q3 denote minimum and maximum values of the dataset within 1.5× IQR from the 25th and 75th percentiles, respectively. Values beyond these upper and lower bounds are outliers represented by dots above or below the whiskers. For confidence intervals, see Supplementary Data File 1. **b**, Average and maximum yield benefit from each year from the optimal dose rate plotted against rainfall during the growing season and during the grain filling period. The effects of DMNB-T6P on yield stimulation (yield potential reflected in maximum yield stimulation achieved data point) and the average stimulation were essentially independent of rainfall amount and timing but more strongly affected by timing of DMNB-T6P application (10 DAA, blue, versus 16 DAA, red). *F* statistics for treatment, genotype, genotype:treatment, respectively, are as follows: 2018: 12.1, 1.83, 0.71; 2020: 3.07, 0.33, 1.68; 2021: 3.23, 11.73, 1.47; 2022: 4.42, 2.57, 1.56, with the corresponding degrees of freedom being 2018: 2, 2, 4; 2020: 2, 2, 4; 2021: 2, 3, 6; 2022 2, 3, 6. df, degrees of freedom; ha, hectare.

Yield in all 4 years at all doses had significance values in ANOVA analyses of *P* = 0.00011, *P* = 0.065, *P* = 0.0348 and *P* = 0.010 for each year, 2018, 2020, 2021 and 2022, respectively (Fig. 2). A combined analysis over all 4 years using a mixed model framework fitted using restricted maximum likelihood (REML) showed a statistical significance of *P* < 0.001 for the effect of DMNB-T6P on yield, grain number and size in the study as a whole (Supplementary Table 3). As farmers would apply an optimal dose in practice, we took optimal dose (dose 2/ 3) to calculate average responses to DMNB-T6P for best prediction of future impact on wheat yields. As a consequence of the 10 DAA application, and considering the *P* < 0.001 for the study as a whole, yields were increased in 2018 by 8.94–17%, average +12.7%; in 2020 by 5–15.4%, average +9.30%; and in 2022 by 6.67–12.6%, average +9.26%. The overall average yield increase for 10 DAA treatment was +10.4%.

Treatment responses were consistent across genotypes (genotype: treatment; Fig. 2). In 2021, after application of DMNB-T6P at 16 DAA instead of 10 DAA, yield was still increased (*P* *=* 0.0348) although by less (1.36–7.52%, average +4.7%) than spray application at 10 DAA. This effectiveness of a later spray at 16 DAA revealed that there is a relatively broad time window (6 d over 10–16 DAA) when a single application of DMNB-T6P increases yield under field conditions. Only in 2021 and 2022 were differences between genotypes observed and also in the trial in Mexico (Extended Data Fig. 2b,c). We attribute these, in 2021, to the delayed 16 DAA application and, in 2022, to a lower response of DM Ceibo +6.67% compared to Saeta +12.6%.

### Response to DMNB-T6P is robust under contrasting rainfall

**Extended Data Fig. 3:**
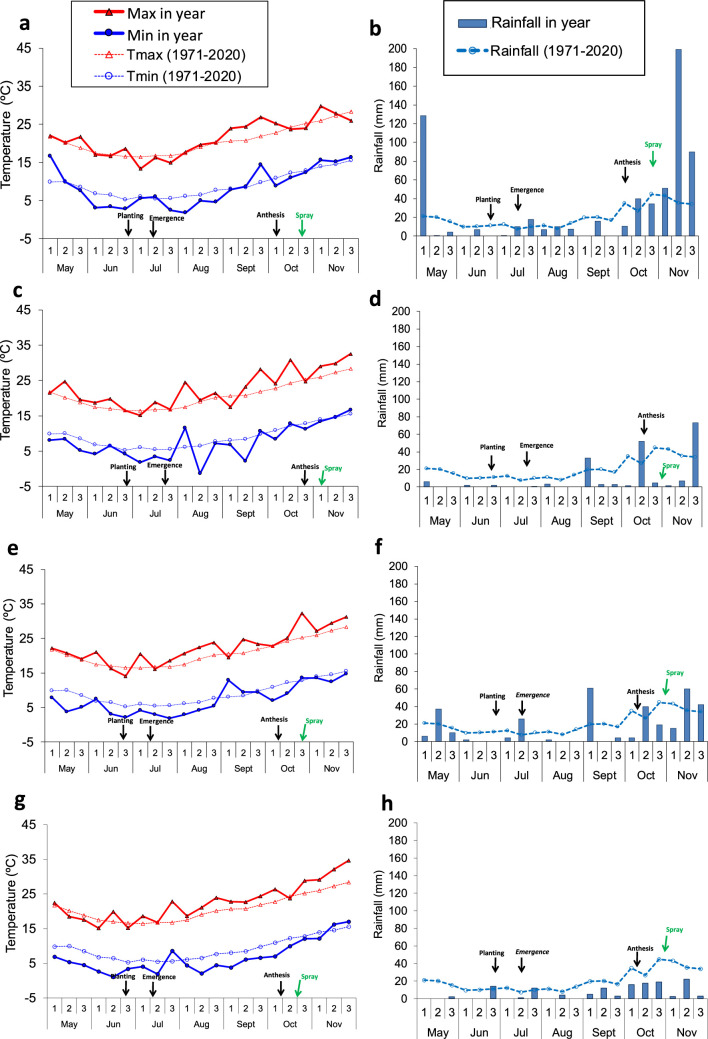
Weather data. Maximum and minimum average temperatures **(a, c, e, g)** and rainfall **(b, d, f, h)** during the wheat cycle in year 1 (2018 **a, b**), year 2 (2020 **c, d**), year 3 (2021 **e, f**) and year 4 (2022 **g, h**) and historical average (1971–2020) at Oliveros Weather Station, Santa Fe, Argentina.

The rainfall in the 4 years of the experiment compared to the historical rainfall averages was highly variable, ranging from 40% above (2018) to 70% below (2020) (Extended Data Fig. 3). Notably, rainfall was the only contributor to water availability; no irrigation was used at any stage. The years 2018 and 2021 had sufficient rainfall to achieve yields of 5.5–6 tons per hectare (noted as ‘wet’ years in Fig. 2), in contrast to ‘dry’ years in 2020 and 2022 where yields were 2.7–3.5 tons per hectare.

The wettest year (+40% above average rainfall, 2018) yielded the highest significance level (*P* = 0.00011) for yield and the greatest magnitude of improvement (+17% for Saeta, +0.88 tons per hectare). A very dry year in 2020 (70% below average rainfall; Extended Data Fig. 3) resulted in the lowest overall yields and showed the lowest overall significance of the four trials as a whole (*P* = 0.065; Fig. 2). There was a yield increase by +15.4% (+0.4 tons per hectare) in MS INTA 415 at dose 2 (*P* = 0.005). Another dry year in 2022 showed yield improvement (*P* = 0.010) between 6.67% and 12.6%, average 9.26%. Notably, when taken together, the average yield increases after 10 DAA application in wet and dry years were similar (+12.7% wet compared to +9.30% dry), demonstrating that signaling-precursor-enabled release of T6P brings similar benefits in the field in both yield potential and yield resilience.

This combination of increases in yield under both good and suboptimal conditions reflects historical improvements in yield through breeding, which has produced germplasm with higher yield under yield potential conditions and stress conditions^27,28^. Comparing our data to current improvements in wheat yield potential—estimated as 0.6% per annum (pa) globally^4^—yield increases (+12.7%) in the observed wet year (2018) represent the equivalent of more than 20 years of advance at current rates of yield potential increase. Even when compared to the national potential for Argentinian wheat of 0.74% pa^29^, this represents more than 17 years worth of increase in yield potential.

### Increased yield is driven by both grain number and weight

**Fig. 3:**
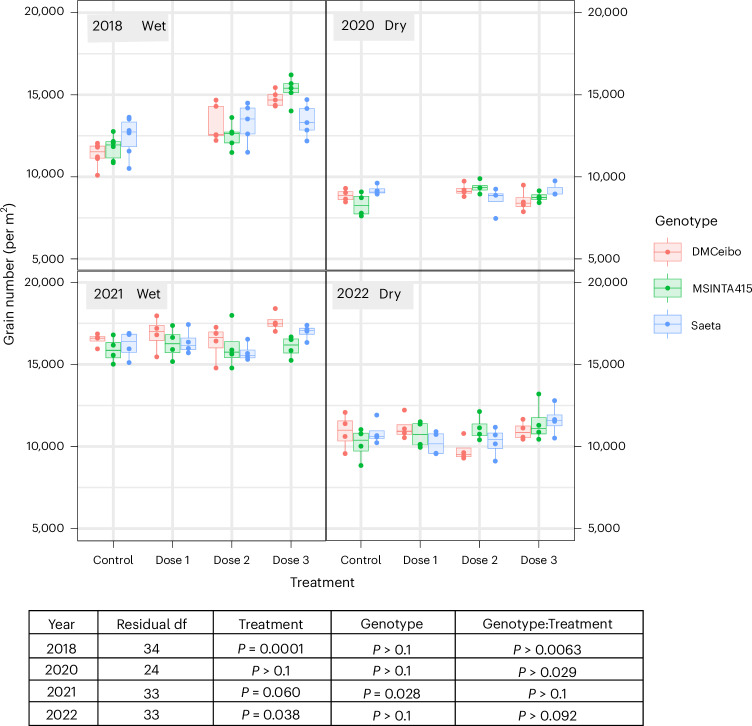
Grain number per m^2^ in response to DMNB-T6P spray over four seasons. Three spring wheat varieties, DM Ceibo, MS INTA 415 and Saeta, grown 2018–2022 at two dose rates of DMNB-T6P in 2018, 2020 and three dose rates of DMNB-T6P in 2021, 2022 compared to control with no DMNB-T6P. Each data point represents an individual plot (*n* = 5 in 2018; *n* = 4 in 2020, 2021 and 2022). Statistical analysis of each Argentinian field trial was performed using a two-way factorial ANOVA accounting for the randomized complete block layout in R version 4.2.1. Additionally, a combined analysis over all 4 years was performed using a mixed model framework fitted using REML (Supplementary Table 3). All data are shown on harmonized scales; for plots with expanded scales, plots with means and comparison of SEDs of combined means and individual means, see also Extended Data Fig. 10. Box plots range from the first quartile (Q1) to the third quartile (Q3) of the distribution and contain the 25th to 75th percentiles of the dataset, respectively, representing the interquartile range (IQR). The center line inside each box represents the median value (50th percentile). Whiskers extending below Q1 and above Q3 denote minimum and maximum values of the dataset within 1.5× IQR from the 25th and 75th percentiles, respectively. Values beyond these upper and lower bounds are outliers represented by dots above or below the whiskers. *F* statistics for treatment, genotype, genotype:treatment, respectively, are as follows: 2018: 32.54, 0.05, 4.31; 2020: 0.66, 0.91, 3.25; 2021: 2.73, 3.98, 0.63; 2022: 3.15, 0.2, 2.01, with the corresponding degrees of freedom being 2018: 2, 2, 4; 2020: 2, 2, 4; 2021: 2, 3, 6; 2022 2, 3, 6. For confidence intervals, see Supplementary Data File 1. df, degrees of freedom.

Grain number is considered the main contributor to wheat yield potential improvement not only in Argentina but also in other world regions^30–32^. Consistent with this, grain number per m^2^ contributed most to our observed yield increases, particularly in 2018 (Fig. 3). Spikelet formation and fertilized florets per spikelet were determined before or at anthesis, respectively^33–35^. We account for the increases in grain number through an increase in grain retention after 10 DAA. Loss of grain can occur after anthesis, particularly under heat and drought^36^. It is noticeable, however, that, even in the wettest year, 2018 (Fig. 3), and under irrigation at CIMMYT (Extended Data Fig. 2b), where drought effects would have been minimal, grain numbers were increased by DMNB-T6P. Effects on grain number may also depend on the number of primary and secondary spikelets formed before treatment. Another possibility, that DMNB-T6P increases tiller and spike survival, we consider less likely as most spikes are lost well before 10 DAA between the onset of stem elongation and anthesis^37^. The numbers of spikes were recorded in 2022; no difference was observed between treatments per unit area.

**Fig. 4:**
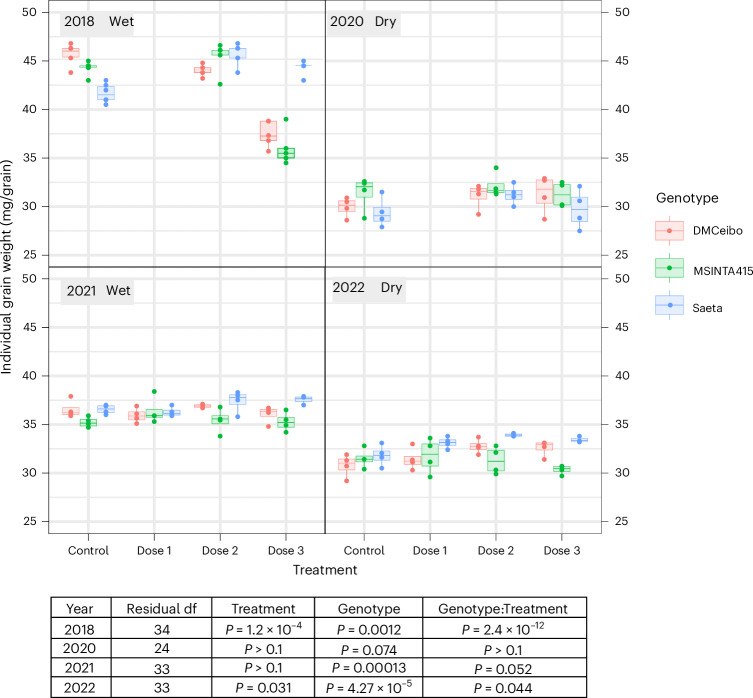
Individual grain weight (mg) in response to DMNB-T6P spray over four seasons. Three spring wheat varieties, DM Ceibo, MS INTA 415 and Saeta, grown in 2018–2022 at two dose rates of DMNB-T6P in 2018, 2020 and three dose rates of DMNB-T6P in 2021, 2022 compared to control with no DMNB-T6P. Each data point represents an individual plot (*n* = 5 in 2018; *n* = 4 in 2020, 2021 and 2022). Statistical analysis of each Argentinian field trial was performed using a two-way factorial ANOVA accounting for the randomized complete block layout in R version 4.2.1. Additionally, a combined analysis over all 4 years was performed using a mixed model framework fitted using REML (Supplementary Table 3). All data are shown on harmonized scales; for plots with expanded scales, plots with means and comparison of SEDs of combined means and individual means, see also Extended Data Fig. 10. Box plots range from the first quartile (Q1) to the third quartile (Q3) of the distribution and contain the 25th to 75th percentiles of the dataset, respectively, representing the interquartile range (IQR). The center line inside each box represents the median value (50th percentile). Whiskers extending below Q1 and above Q3 denote minimum and maximum values of the dataset within 1.5× IQR from the 25th and 75th percentiles, respectively. Values beyond these upper and lower bounds are outliers represented by dots above or below the whiskers. *F* statistics for treatment, genotype, genotype:treatment, respectively, are as follows: 2018: 94.94, 8.27, 39.55; 2020: 2.13, 2.91, 0.47; 2021: 0.77, 11.94, 2.37; 2022: 3.34, 13.86, 2.47, with the corresponding degrees of freedom being 2018: 2, 2, 4; 2020: 2, 2, 4; 2021: 2, 3, 6; 2022 2, 3, 6. For confidence intervals, see Supplementary Data File 1. df, degrees of freedom.

**Extended Data Fig. 4:**
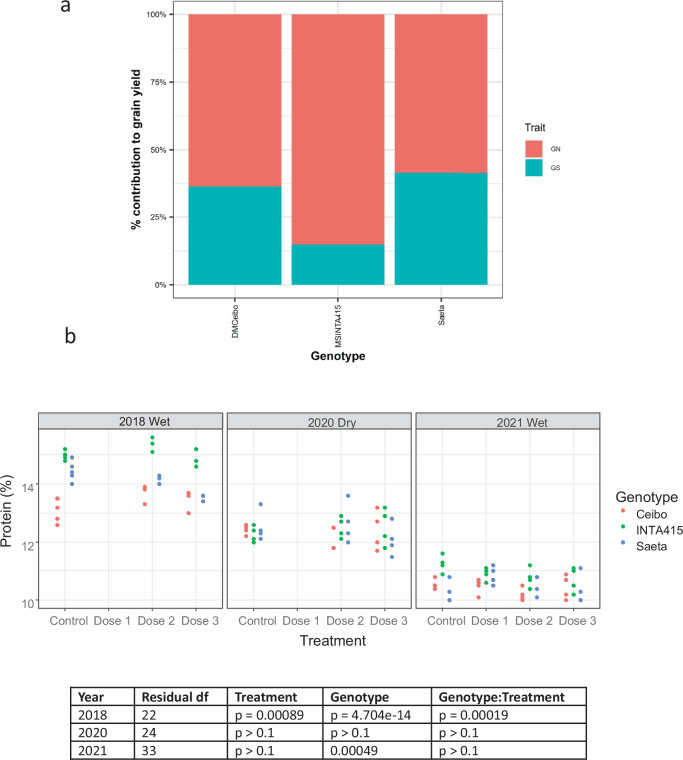
Further data from trial in Argentina. (**a**) The percentage contributions of grain number and grain size to yield increase of DMNB-T6P treatment compared to control from the field trials in Argentina 2018, 2020, 2021, 2022. Calculated as % grain number increase/ % grain yield increase x 100 and % grain size increase/ % grain yield increase x 100 as overall average for each genotype. (**b**) Protein content % in response to DMNB-T6P spray. Three spring wheat varieties DM Ceibo, MS INTA 415 and Saeta grown 2018–2021 at two dose rates of DMNB-T6P in 2018, 2020 and three dose rates of DMNB-T6P in 2021 compared to control with no DMNB-T6P. Each data point represents an individual plot. F statistics: 2018: 9.84, 168, 8.97; 2020: 0.14, 0.18, 1.08; 2021: 1.71, 9.69, 1.77. Degrees of freedom: 2018: 2, 2, 4; 2020: 2, 2, 4; 2021: 2, 3, 6.

Individual grain weight also contributed to yield increase (Fig. 4) in both wet (2018) and dry (2022) years. Interestingly, we did not observe a wide-ranging tradeoff between grain number and grain weight, an effect that can confound genetic attempts to increase one or the other^38^, except in 2018 in MS INTA 415 and DM Ceibo where the large increase in grain numbers in response to DMNB-T6P may have restricted subsequent grain filling (as also seen for the KAUZ*Z/MNV/KAUZ variety in Mexico; Extended Data Fig. 2b,c). Otherwise, grain number and weight notably increased together, with a tendency for Saeta to increase grain weight the most (grain weight contributed 41% of yield increase compared to 35% in Ceibo and 14% in MS INTA). Accordingly, MS INTA 415 increased grain number the most, contributing 86% of yield increase (Figs. 3 and 4 and Extended Data Fig. 4a). Heavier grains without great changes in grain number per m^2^ have been associated with prior yield improvements in Australia, Mexico and the Hebei province of China^39–41^. This observed ability of T6P signaling precursor to enhance both grain number and grain size together indicates broad potential in wheat yield improvement over geographical regions where grain number or size improvement may be favored and may break the recalcitrant tradeoff between grain size and number.

### Yield is increased sustainably with maintained grain protein

**Fig. 5:**
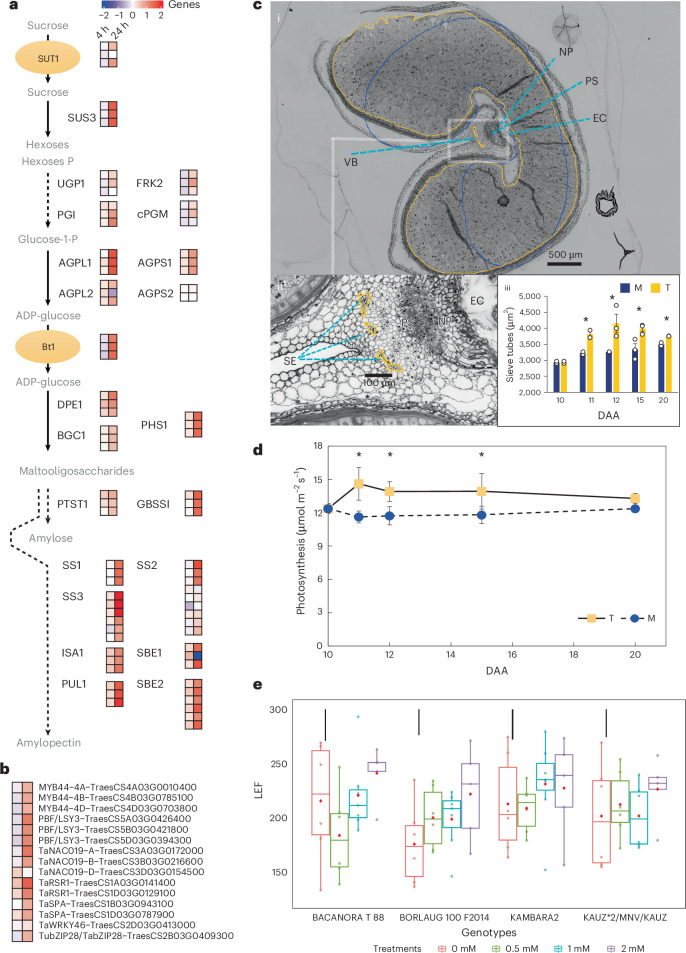
Sink and source both increase in higher yielding wheat when treated with DMNB-T6P at 10 DAA. **a**, Pathway of starch synthesis in wheat endosperm. Gene expression dynamics of enzymes and transporters from sucrose import to starch synthesis in wheat endosperm^45^. Metabolites are in gray letters, enzymes in black. log_2_FC of gene expression from four biological replicates per condition at 4 h (left-hand column of squares) and at 24 h (right-hand column of squares) comparing DMNB-T6P treatment with control. Values of gene expression are colored from blue to red. All log_2_FC values are capped to ±2. Dashed lines indicate multiple steps. AGPL/S, ADP-glucose pyrophosphorylase large/small subunit; BGC1, B-granule content 1; Bt, ADP-glucose brittle-1 transporter; cPGM, cytoplastic phosphoglucomutase; DPE, disproportionating enzyme; FRK, fructokinase; GBSS, granule bound starch synthase; ISA, isoamylase; PFK, phosphofructokinase; PGI, glucose-6 phosphate isomerase; PHS1, plastidial α-glucan phosphorylase; PTST1, protein targeting to starch; PUL, pullulanase; SBE, starch branching enzyme; SS, soluble starch synthase; SUS, sucrose synthase 3; SUT, sucrose transporter; UGP, UDP-glucose pyrophosphorylase. α-Glc, glucose; Frt, fructose (*n* = 4). **b**, Expression of transcription factors^46^ regulating starch synthesis during wheat endosperm development at 4 h (left-hand column of squares) and at 24 h (right-hand column of squares) comparing DMNB-T6P treatment with control (*n* = 4). **c**, (i) Transverse section at 20 DAA of DMNB-T6P-treated grain, with starchy endosperm and sieve tube area silhouetted in orange. Overlayed, control starchy endosperm area in blue. EC, endosperm cavity; NP, nuclear projection; PS, pigment strand; SE, sieve elements; VB, vascular bundle. (ii) Inset shows section of sieve tubes. (iii) The dynamics of sieve tube area increases (µm^2^) 11–20 DAA, *n* = 3. T, DMNB-T6P treated; M, mock/control/untreated; * = Day 11 *P* = 0.048, Day 12 *P* = 0.045, Day 15 *P* = 0.025, Day 20 *P* = 0.060, pairwise *t*-test with s.e.m. shown as error bars. **d**, Net photosynthesis of the flag leaf 10–20 DAA, *n* = 4. T, DMNB-T6P treated; M, mock/control/untreated; * = Day 11 *P* = 0.033, Day 12 *P* = 0.037, Day 15 *P* = 0.035, pairwise *t*-test with s.e.m. shown as error bars. **e**, Rates of LEF in flag leaves measured 13 DAA in four genotypes (BACANORA T 88, BORLAUG100 F2014, KAMBARA2, KAUZ*2/MNV/KAUZ) treated with three concentrations of DMNB-T6P (0.5 mM, 1 mM and 2 mM DMNB-T6P compared to control (0 mM)) at the experimental field station, CENEB, in Ciudad Obregon, Sonora, Mexico. Each dosage in the field contains six observations per genotype (*n* = 24), ANOVA *P* = 0.018. SEDs of the means (red diamonds) of different treatments within a genotype are shown above as black lines. Box plots range from the first quartile (Q1) to the third quartile (Q3) of the distribution and contain the 25th to 75th percentiles of the dataset, respectively, representing the interquartile range (IQR). The center line inside each box represents the median value (50th percentile). Whiskers extending below Q1 and above Q3 denote minimum and maximum values of the dataset within 1.5× IQR from the 25th and 75th percentiles, respectively. Values beyond these upper and lower bounds are outliers represented by dots above or below the whiskers.

Grain protein content (%) was largely unchanged and even increased in the higher yielding crop (Extended Data Fig. 4b). In 2018, when grain yield was increased by 17% in Saeta, protein was decreased by a small amount on this occasion, likely because of the large increase in grain weight (Figs. 4 and 5). In the same year, there were increases in protein content for DM Ceibo and MS INTA 415 (Extended Data Fig. 4b). Because fertilizer applications were unchanged, this suggested that nitrogen uptake from soil may have increased together with enhanced nitrogen metabolism to support higher yield. SnRK1, a primary target of T6P, was shown to coordinate carbon and nitrogen metabolism^42,43^; the known action upon SnRK1, therefore, likely drives these observed improved nitrogen use efficiencies.

**Extended Data Fig. 5:**
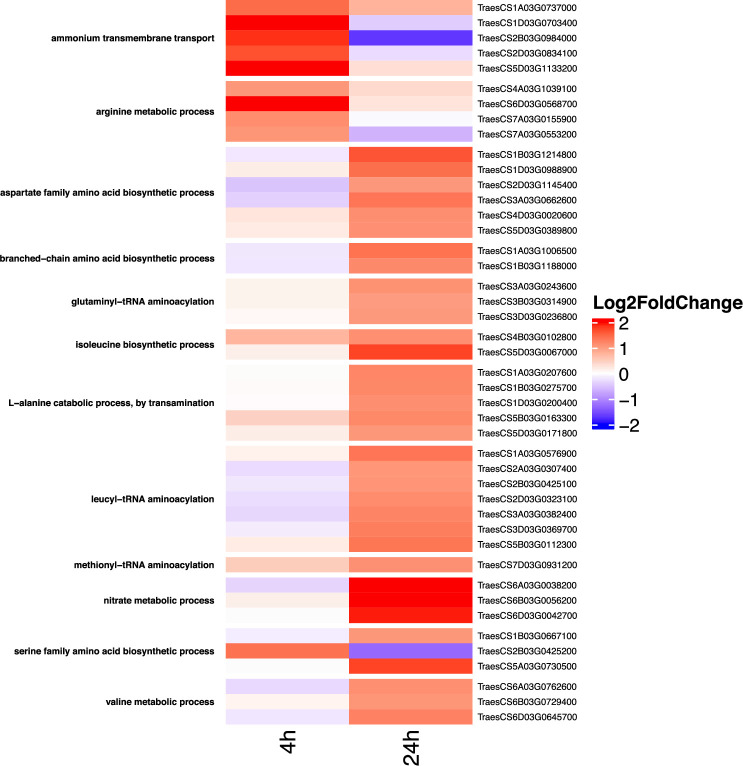
Gene expression of nitrogen-related genes in wheat. Data annotated according to Chinese Spring IWGSC RefSeq v1.1 reference genome assembly [Ensembl Plants; https://plants.ensembl.org/Triticum_aestivum/Info/Index; Yates A. D. et al. Ensembl Genomes 2022: an expanding genome resource for non-vertebrates. *Nucleic Acids Research* **50**, D996–D1003 (2022). 10.1093/nar/gkab1007], 4 and 24 h after DMNB-T6P treatment compared to control. Log_2_-fold change from four biological replicates per condition. Log_2_-fold values are capped to ±2. Ammonium transmembrane transport, arginine metabolic processes, aspartate family amino acid biosynthetic process, branched amino acid biosynthetic process, glutaminyl-tRNA aminoacylation, isoleucine biosynthetic process, L-alanine transamination, leucyl-tRNA aminoacylation, methionyl-tRNA aminoacylation, nitrate metabolic process, serine family amino acid biosynthetic process, valine metabolic process.

As a consequence, the yield per unit of fertilizer application was increased upon treatment with DMNB-T6P. The synthetic nitrogen fertilizer supply chain generated estimated emissions of 1.13 GtCO_2_ in 2018 (ref. ^43^), representing 2.1% of all global greenhouse gas emissions. Nitrogen fertilizer also generates N_2_O as a by-product of soil microbial metabolism, a greenhouse gas with 265 times more global warming potential than CO_2_. Our results revealed that DMNB-T6P application increased yield sustainably, thereby breaking the link between increased yield and need for fertilizer application (and, hence, associated greenhouse gas emissions). RNA sequencing (RNA-seq) data from grain 24 h after treatment with DMNB-T6P revealed upregulation of several genes associated with amino acid biosynthesis (aspartate family, branched amino acid, isoleucine, serine and valine) and aminoacyl-tRNA synthetases (Extended Data Fig. 5), all of which play central roles in protein synthesis^44^.

In economic terms, estimates of the final cost of synthesis of DMNB-T6P are set to be in the region $300 per ton, which equates to a few cents per hectare at current rates of application. If this stimulated yield by the average of 10.4% that we observed from field trials (wet and dry years at 10 DAA application), then the increase in global wheat yield average of 3.6 tons per hectare would be 0.37 tons per hectare (worth an extra $116 per hectare (wheat price in February 2023)); this amounts to $25.6 billion globally (221 million hectares grown in 2021). With further improvements in formulation and adjuvants, it may be possible to reduce application dose rate and/or exceed the yield improvements presented here.

### Carbon ‘source’ and ‘sink’ are both enhanced to increase yield

To gain mechanistic insight into how DMNB-T6P increases yield in wheat (predominantly starch), both grain (RNA-seq gene expression and structural analysis) and flag leaves (CO_2_ uptake and linear electron flow (LEF)) were examined after treatment. Together, these revealed striking wide-scale stimulation of starch biosynthesis.

**Extended Data Fig. 6:**
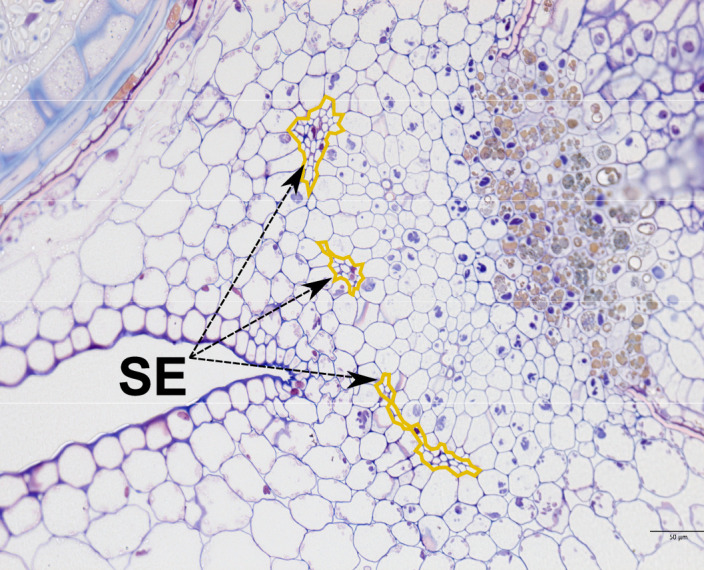
Higher magnification x 20 of transverse section at 20 DAA of DMNB-T6P treated grain. From Fig. 4cii, SE = sieve elements.

DMNB-T6P increased gene expression from sucrose transport into grain (*TaSUT1*) and the pathway to starch^45^ as well as associated transcription factors^46^ that regulate starch biosynthesis (Fig. 5a,b). In particular, these included upregulation of genes associated with key, flux-associated steps (Fig. 5a): sucrose breakdown (sucrose synthase, *TaSUS3*); interconversion of glucose-1-phosphate (G1P) to ADP-glucose (ADPG) (by ADP-glucose pyrophosphorylase large subunit, *TaAGPL1*); import of ADPG into plastids through its dedicated transporter (ADP-glucose brittle-1, *TaBt1*) as well as diverse steps catalyzing synthesis of both amylose and amylopectin as primary starch components (plastidial α-glucan phosphorylase *TaPHS1* and granule bound starch synthase *GBSSI*; soluble starch synthases (*TaSS1*, *TaSS2* and *TaSS3*); and isoamylase *TaISA1*, pullulanase *TaPUL1* and starch branching enzyme *TaSBE2*). This observed, broad enhancement of ‘sink’ strength was further confirmed by sectioned grain that showed increased endosperm volume upon treatment with DMNB-T6P and areas of sieve tube elements in the vascular bundles that supply assimilates to grain (Fig. 5c and Extended Data Fig. 6). Although it was noted that wheat phloem servicing grain development has spare transport capacity^47,48^, whether the corresponding enhanced sieve tube development is necessary for enhanced sucrose transport will need to be established.

**Extended Data Fig. 7:**
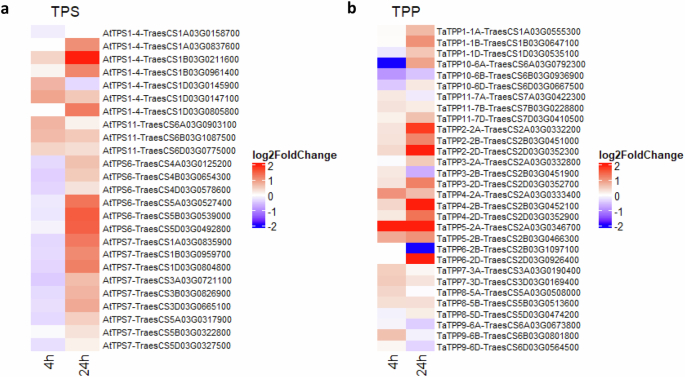
Gene expression of trehalose phosphate synthases and phosphatases. **(a)** Trehalose phosphate synthases in wheat grain [annotated according to *Plant Physiology* **177**, 12–23 (2018), 10.1104/pp.17.01634] 4 and 24 h after DMNB-T6P treatment compared to control. Log_2_fold change from four biological replicates per condition. Log_2_fold values are capped to ±2. **(b)** Gene expression of trehalose phosphate phosphatases in wheat grain [annotated according to *BMC Plant Biology* **22**, 120 (2022), 10.1186/s12870-022-03504-0], 4 and 24 h after DMNB-T6P treatment compared to control. Log_2_fold change from four biological replicates per condition. Log_2_fold values are capped to ±2.

**Extended Data Fig. 8:**
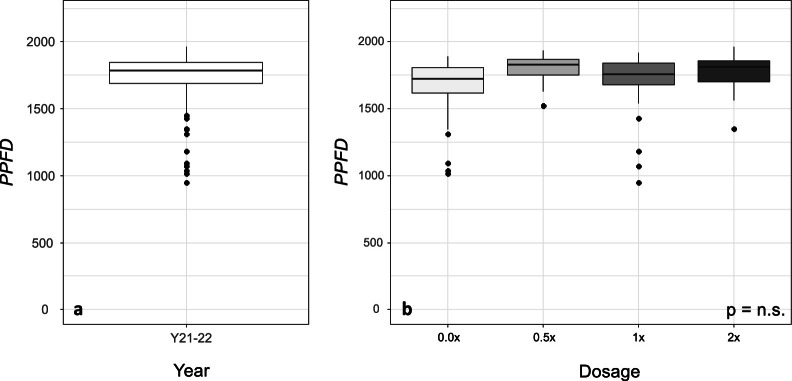
Chl fluorescence ambient photosynthetic photon flux density (PPFD). Measurements are indicated by **(a)** year and **(b)** dosage. There was no significant difference between treatments for ambient PPFD at the time of measurement, indicating that differences in light intensity are not a contributing factor to differences seen between genotypes or treatments. Box plots range from the first (Q1) to the third quartile (Q3) of the distribution and contain the 25th to 75th percentiles of dataset, respectively, representing the interquartile range (IQR). The centre line inside each box represents the median value (50th percentile). Whiskers extending below Q1 and above Q3 denote minimum and maximum values of the dataset within 1.5 x IQR from the 25th and 75th percentiles, respectively.

Assessment of the ‘source’ in flag leaves revealed that CO_2_ fixation was increased in plants with spikes treated only (*P* < 0.05; Fig. 5d) together with LEF in plants in the field (*P* < 0.018; Fig. 5e and Extended Data Fig. 8). LEF vitally generates ATP and NADPH to drive the Calvin cycle^49^. Together, these data show that DMNB-T6P induces large increases in the source-to-sink process for yield. The breadth of the effects was further supported by observed wide-ranging transcriptional effects in the T6P pathway across trehalose phosphate synthase and trehalose phosphate phosphatase genes (Extended Data Fig. 7a,b).

### Yield is increased in other cereals

**Extended Data Fig. 9:**
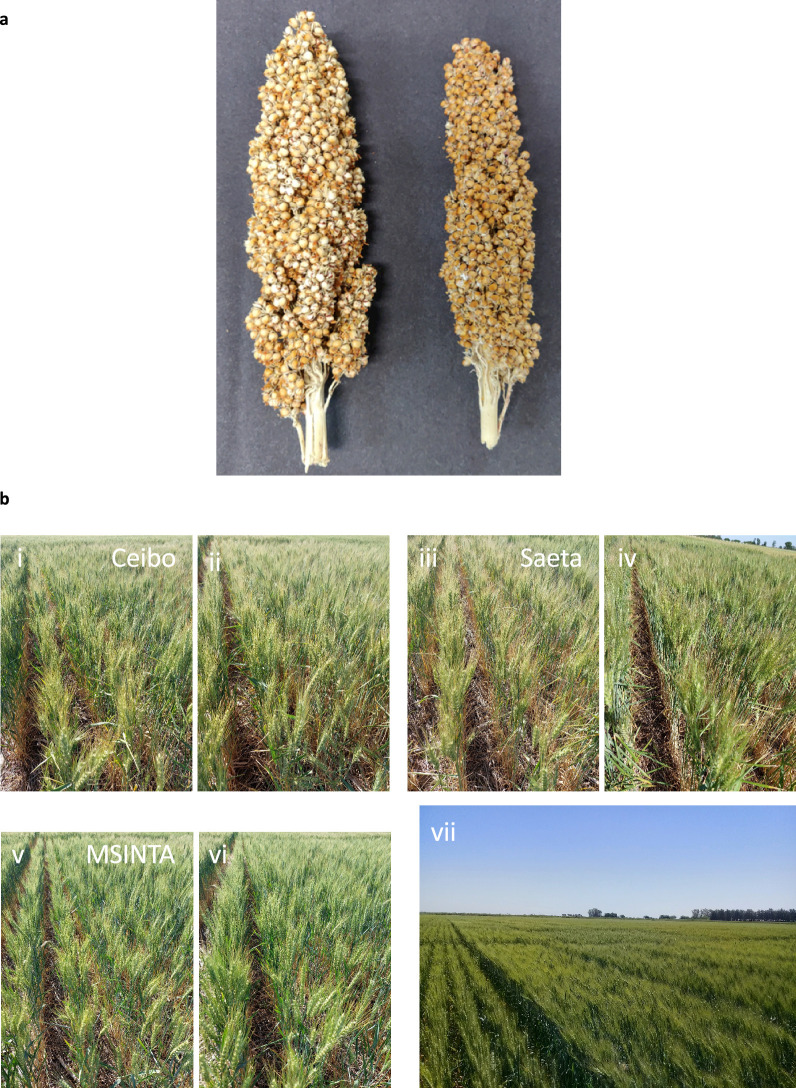
Additional photographic data. **(a)** Sorghum treated with DMNB-T6P to reproductive structures. 8 ml per spike of 2 mM DMNB-T6P spray applied at 7 and 14 DAA (left) compared to spray with no DMNB-T6P (right). Plants were fully hydrated until anthesis when exposed to drought to 60% of pot weight until harvest. **(b) i-vi**: Wheat growing at National Institute of Agricultural Research (INTA) Oliveros Research Station, Santa Fe Argentina. DM Ceibo, Saeta and MS INTA 415 spring in 2022 at 20 DAA: **i, iii, v** control plots; **ii, iv, vi** plots sprayed with dose 3 of DMNB-T6P. **vii** Landscape view from 2021 at 15 DAA.

The T6P pathway is widespread and found in other crops. To assess the potential for expansion into the field for other crops, we also treated sorghum and barley with microdoses of DMNB-T6P in a controlled environment in both well-watered conditions and under conditions of drought implemented from anthesis until harvest. Yield was increased in both crops under both conditions by +10.8% to +24.3% (Supplementary Table 4). Sorghum displayed a visible effect of DMNB-T6P on yield (Extended Data Fig. 9a). Given that we now show here that data from prior wheat treatment in a controlled environment^23^ are translated to observed effects in the field, these additional controlled environment data for other important cereals excitingly suggest wider applicability of the technology in farming.

## Discussion

Wheat is a crop that is limited by both ‘source’^50^ and ‘sink’^51^. Current strategies typically target source (for example, improved photosynthesis) or sink (for example, increased grain number and fruiting efficiency) traits separately, largely through gene-focused methods. Now, here, we suggest that is it possible to overcome the limitations of source and sink concurrently using chemical methods and that this drives robust beneficial responses under field conditions.

Prior suggested efficacy of the signaling precursor DMNB-T6P in boosting wheat yield in controlled environment conditions^23^ has now been translated to a range of realistic and contrasting environments. This demonstrates that a single well-timed foliar microdose of DMNB-T6P promotes yield potential. It also improves yield under low and varying rainfall, as is necessary to minimize crop losses due to drought and ongoing climate change.

The mechanistic analyses of these benefits reveal that application of DMNB-T6P during early grain filling strongly induces the plant’s capacity to synthesize starch—the world’s most important food carbohydrate staple. The period from 10 DAA coincides with grain yield potential transitioning from being source limited to sink limited, as grain development shifts from pre to fill phase^51^. T6P likely enhances this increase in ‘sink’ capacity through broad upregulation of transcription factors and key regulatory steps ranging from sucrose transport through to starch synthesis. Observed increased vascular connections in the grain as well as enhanced flag leaf photosynthesis appear to have combined upon treatment to link enhanced ‘source’ with enhanced ‘sink’. Upregulation of genes for amino acid and protein synthesis in grain may have also increased sink strength for nitrogen. Photosynthesis is increased 11–20 DAA in response to enhanced grain sink strength^50^ for a period long enough to supply starch synthesis in the grain yet short enough to limit any water loss penalty through greater stomatal opening that could accompany photosynthetic enhancement throughout growth^52^. The later harvest in 2018, 2020 and the longer period of maturation did not affect yield in those years; yield benefits may be achieved largely between 10 DAA and 20 DAA. Although field-level DMNB-T6P application is to the whole canopy—leaves and spikes—we suggest that effects achieved on the whole source–sink system are sink-led from the spike through sink demand^22^ that leads to enhanced photosynthesis and sucrose transport from the flag leaf^50^ because effects on yield and photosynthesis (Fig. 5d,e) are similar when only spikes are treated with DMNB-T6P^23^.

A tradeoff between grain size and number has often confounded attempts to increase either through breeding or molecular intervention^53^. DMNB-T6P in many cases increased both grain size and number together, especially for varieties Saeta and Ceibo (Figs. 3 and 4 and Extended Data Fig. 4a). Further detailed analysis may enable elucidation of genes and mechanisms that will promote both grain size and number. Topical applications of DMNB-T6P applied at earlier development times might further increase grain yield by promoting branching (tillers, spikelets and florets^54,55^) and blocking abortion/abscission (florets and grains^56^).

This T6P signaling precursor strategy has enabled yield increases, in excess of historical selection and of current breeding, and beyond the level to which the T6P pathway has contributed to yield improvements through unconscious selection of the T6P pathway through breeding^57^. Moreover, the apparently broad window of application time in a manner requiring no specialist equipment and in a formulation that is tank mixable, and so can be used as a co-spray with good storage properties, renders this a highly practical approach.

Currently, it is a challenge to perturb crop physiology through genetic or other means to promote yield-promoting responses that strongly elevate both sink capacity and source or, indeed, whole primary metabolic pathways. Gene editing methods, which can target only a few genetic elements, have been limited to disease control and quality traits^58^. It should be noted that many previous technologies and strategies for yield improvement have provided promise in controlled environments but then eventually do not deliver benefit in the field^15^. Elevating the source-to-sink pathway benefits both yield potential and yield under low and variable rainfall that typifies global agriculture.

DMNB-T6P increased yield per unit of fertilizer—our method uses only microdoses (grams) compared to routine use of kilograms of nitrogen fertilizer—and resulted in upregulation of amino acid and protein synthesis in the grain. This breaks the currently presumed necessary ‘fertilizer-for-yield’ chain. We think that this approach may, therefore, allow a step change in the development of a new technology to enhance wheat yields with wide potential ramifications for improving supply of a major food staple and potentially other crops in a sustainable way that avoids the consequences of future fertilizer use.

## Methods

### Optimized DMNB-T6P synthesis

#### General chemical materials and methods

All reagents were purchased from commercial sources and were used without further purification unless noted. Molecular sieve (4 Å, powder) used in reactions was activated at 350 °C for more than 12 h. Dry solvents for reactions were purchased from Sigma-Aldrich; the following abbreviations are used: PE, petroleum ether (boiling point (bp) 40–60 °C); EtOAc, ethyl acetate; THF, tetrahydrofuran. Thin layer chromatography (TLC) was carried out using Merck aluminium-backed sheets coated with Kieselgel 60-F_254_ silica gel. Visualization of the reaction components was achieved using UV fluorescence (254 nm) and/or by charring with an acidified *p*-anisaldehyde solution in ethanol. Organic solvents were evaporated under reduced pressure, and the products were purified by flash column chromatography on silica gel (230–400 mesh). Proton nuclear magnetic resonance (^1^H NMR) spectra were recorded on Bruker AVG400, AVH400 or AVB400 (400 MHz) spectrometers, and the chemical shifts are referenced to residual CHCl_3_ (7.26 ppm, CDCl_3_), CHD_2_OD (3.30 ppm, CD_3_OD) and C_6_HD_5_ (7.16 ppm, C_6_D_6_). Carbon nuclear magnetic resonance (^13^C NMR) spectra were recorded on Bruker AVG400 (100 MHz) spectrometers and are proton decoupled, and the chemical shifts are referenced to CDCl_3_ (77.0 ppm) or CD_3_OD (49.0 ppm). Assignments of NMR spectra were based on two-dimensional experiments (1H-1H COSY, DEPT-135, HSQC and HMBC) if required. Chemical shift for ^31^P NMR is reported with reference to phosphoric acid (0.00 ppm). Reported splitting patterns are abbreviated as follows: s, singlet; d, doublet; t, triplet; q, quartet; p, pentet; hept, heptet; m, multiplet; br, broad. Low-resolution mass spectra (LRMS) were recorded on a Micromass Platform 1 spectrometer using electrospray ionization (ESI) or on a Bruker Daltronic MicroTOF spectrometer. High-resolution mass spectra (HRMS) were recorded on a Bruker Daltronic MicroTOF spectrometer using ESI (*m*/*z* values are reported in Daltons). Optical rotations were measured on a PerkinElmer 241 polarimeter at 589 nm (Na D-line) with a path length of 1.0 dm at ambient temperature and are in units of degree ml g^−1^ dm^−1^. Infrared spectra were recorded on a Bruker Tensor 27 Fourier Transform spectrophotometer using attenuated total reflectance (ATR), and absorption maxima (*ν* max) are reported in wavenumbers (cm^−1^). X-ray powder diffraction was recorded on a PANalytical Empyrean Series 2 powder diffractometer.

#### Preparation of K_2_CO_3_ solution for deprotection under anhydrous conditions

Anhydrous K_2_CO_3_ (solid, 50 mg, 0.1% (w/v) to methanol) was added into dry methanol (50 ml) under inert condition, and the mixture was stirred at room temperature for 30 min, followed by the addition of dry CH_2_Cl_2_ (10 ml, 20% (v/v) to methanol). The resulting solution was used directly for selective deprotection of **1** (2 g) at cold temperature without filtration. Concentration proves important to selectivity, and dosage must be increased proportionally for large-scale reactions.

##### 2,3,4,6,2′,3′,4′,6′-Octakis-O-(trimethylsilyl)-d-trehalose (**1**)



Under inert atmosphere (argon), to a stirred solution of d-(+)-trehalose dihydrate (25.00 g, 66.08 mmol) in dry pyridine (250 ml) were added chlorotrimethylsilane (100.64 ml, 792.96 mmol) and hexamethyldisilazane (110.23 ml, 528.64 mmol) successively at cold temperature (ice water mixture, 2–4 °C), and the resulting mixture was allowed to warm to room temperature. After stirring for 6 h, the thick solution was concentrated under vacuum, and the crude residue was suspended in CH_2_Cl_2_ (300 ml) and washed with saturated NaCl (aq.) solution (300 ml). The organic layer was separated; the aqueous layer was extracted with CH_2_Cl_2_ (150 ml × 3); and the combined organic layers were dried over Na_2_SO_4_, filtered and concentrated and dried in vacuum to give the desired compound **1** (60.75 g, quant.) as an amorphous white solid: *R*_*f*_ = 0.68 (PE–EtOAc, 20:1); melting point (mp) 81–82 °C; [α]_D_^25^ + 96.9 (*c* 1.0, CH_2_Cl_2_); literature (lit.) mp 80–82 °C; [α]_D_^25^ + 94 (*c* 1.5, CHCl_3_); ^1^H NMR (400 MHz, CDCl_3_): *δ* 4.91 (d, *J*_1,2_ = 3.1 Hz, *J*_1′,2′_ = 3.1 Hz, 2H, H-1, H-1′), 3.88 (t, *J* = 9.0 Hz, 2H), 3.78 (ddd, *J* = 9.4 Hz, *J* = 4.0 Hz, *J* = 2.4 Hz, 2H), 3.69 (dd, *J* = 11.3 Hz, *J* = 2.4 Hz, 2H), 3.65 (dd, *J* = 11.3 Hz, *J* = 4.0 Hz, 2H), 3.43 (t, *J* = 9.0 Hz, 2H), 3.38 (dd, *J* = 9.4 Hz, *J* = 3.1 Hz, 2H), 0.139 (s, 18H), 0.135 (s, 18H), 0.11 (s, 18H), 0.09 (s, 18H) ppm.

##### 2,3,4,2′,3′,4′,6′-Heptakis-O-(trimethylsilyl)-d-trehalose (**2a**) and 2,3,4,2′,3′,4′-hexakis-O-(trimethylsilyl)-d-trehalose (**2b**)



Potassium carbonate (165 mg, 1.19 mmol) was added into methanol (165 ml, high-performance liquid chromatography (HPLC) grade, 5.5 ml g^−1^); the resulting suspension was stirred at room temperature for 30 min, and then CH_2_Cl_2_ (33 ml, HPLC grade, 1.1 ml g^−1^) was added, followed by the addition of the substrate **1** (30 g, 32.62 mmol, ground, white powder) in one portion. After stirring for 2 h at room temperature, the clear solution was quenched by acetic acid (136.7 μl, 2.39 mmol) and pyridine (193 μl, 2.39 mmol) successively. After removal of the solvent under vacuum, the crude residue was then suspended in CH_2_Cl_2_ (200 ml) and washed with saturated NaCl solution (200 ml), and the organic layer was separated; the aqueous layer was extracted with CH_2_Cl_2_ (50 ml × 3); and the combined organic layers were dried over Na_2_SO_4_, filtered and concentrated and dried under high vacuum overnight, giving a mixture of **2a** and **2b** (25.7 g, quant., **2a**:**2b** = 1:4) as a white foam, which was used directly in the phosphorylation.

**2a** when isolated is a colorless syrup: *R*_*f*_ = 0.21 (PE–EtOAc, 20:1); [α]_D_^25^ + 96.4 (*c* 1.0, CH_2_Cl_2_); lit. [α]_D_^25^ + 113 (*c* 2.5, PE); ^1^H NMR (400 MHz, CDCl_3_): *δ* 4.93 (d, *J*_1′,2′_ = 3.1 Hz, 1H, H-1′), 4.88 (d, *J*_1,2_ = 3.1 Hz, 1H, H-1), 3.91–3.82 (m, 3H), 3.79 (ddd, *J* = 9.4 Hz, *J* = 4.6 Hz, *J* = 2.0 Hz, 1H), 3.74–3.63 (m, 4H), 3.48–3.38 (m, 4H), 1.75 (br s, 1H), 0.16 (s, 9H), 0.140 (s, 9H), 0.138 (s, 18H), 0.12 (s, 9H), 0.11 (s, 9H), 0.10 (s, 9H) ppm.

**2b** when isolated is an amorphous white solid: *R*_*f*_ = 0.48 (PE–EtOAc, 3:1); mp 115–116 °C; [α]_D_^25^ + 99.8 (*c* 1.0, CH_2_Cl_2_); lit. mp 114–115 °C; [α]_D_^22^ + 99.5 (*c* 2.7, CHCl_3_); ^1^H NMR (400 MHz, CDCl_3_): *δ* 4.90 (d, *J*_1,2_ = 3.1 Hz, *J*_1′,2′_ = 3.1 Hz, 2H, H-1, H-1′), 3.89 (t, *J* = 9.0 Hz, 2H), 3.85 (dt, *J* = 9.5 Hz, *J* = 3.2 Hz, 2H), 3.74–3.66 (m, 4H), 3.48 (t, *J* = 9.1 Hz, 2H), 3.42 (dd, *J* = 9.3 Hz, *J* = 3.1 Hz, 2H), 1.73 (br s, 2H), 0.16 (s, 18H), 0.14 (s, 18H), 0.12 (s, 18H) ppm.

##### Synthesis of 4,5-dimethoxy-2-nitrobenzaldehyde (**3**)



Nitric acid (100 ml, 70%) was cooled by an ice water bath (2–4 °C) for 30 min; veratraldehyde (20 g, 120.35 mmol, ground) was added portion-wise with stirring; and the mixture was brought to 10 °C and stirred until a clear solution was obtained (around 1 h). Then, the mixture was poured into an ice water mixture (1,000 ml) while stirring vigorously. The resultant yellow solid was collected by filtration and washed with cold water to remove nitric acid completely, and the solid was recrystallized from boiling ethanol (300 ml), affording **3** (20 g, 79%) in the form of yellow needle crystals: *R*_*f*_ = 0.56 (PE–EtOAc, 3:1); mp 131–132 °C; ^1^H NMR (400 MHz, CDCl_3_): *δ* 10.45 (s, 1H, C*H*O), 7.62 (s, 1H, H-3), 7.42 (s, 1H, H-6), 4.04 (s, 3H, OC*H*_*3*_), 4.03 (s, 3H, OC*H*_*3*_) ppm.

##### 4,5-Dimethoxy-2-nitrobenzyl alcohol (**4**)



Sodium borohydride (3.8 g, 100.6 mmol) was added to an ice-cooled solution of 4,5-dimethoxy-2-nitrobenzaldehyde (**3**) (17.7 g, 83.8 mmol) in anhydrous tetrahydrofuran (THF) (400 ml), and the mixture was stirred at 2–4 °C for 3 h. The reaction was quenched by addition of water (400 ml); the organic layer was separated; and the aqueous layer was extracted with CH_2_Cl_2_ (150 ml × 3). Then, the combined organic layers were dried over anhydrous Na_2_SO_4_ and filtered and concentrated to dryness to give alcohol **4** (17.8 g, quant.) as an amorphous yellow solid: *R*_*f*_ = 0.24 (PE–EtOAc, 3:1); mp 151–152 °C; ^1^H NMR (400 MHz, CDCl_3_): *δ* 7.71 (s, 1H, H-3), 7.18 (s, 1H, H-6), 4.96 (d, *J* = 6.5 Hz, 2H, ArC*H*_*2*_OH), 4.01 (s, 3H, OC*H*_*3*_), 3.96 (s, 3H, OC*H*_*3*_), 2.60 (t, *J* = 6.5 Hz, 1H, O*H*) ppm.

##### Bis-(4,5-dimethoxy-2-nitrobenzyl)-N,N-diisopropylphosphoramidite (**6**)



Under inert atmosphere (argon), to a stirred solution of phosphorus trichloride (13.09 ml, 150 mmol) in dry THF (400 ml) were added diisopropylethylamine (52.25 ml, 300 mmol) and diisopropylamine (42.05 ml, 300 mmol) successively at cold temperature (ice water bath, 2–4 °C). After stirring for 4 h at the same temperature, the suspended solution was cooled to −15 °C. Then, triethylamine (46.00 ml, 330 mmol) and 4,5-dimethoxy-2-nitrobenzyl alcohol (**4**) (64.0 g, 300 mmol) were added successively. The resulting mixture was allowed to warm to room temperature and stirred for a further 20 h in the dark. Saturated NaHCO_3_ (aq.) solution (200 ml) was added, and the resulting suspension was filtered, washed with water (50 ml × 2) and CH_3_CN (50 ml × 2) and completely dried under vacuum to give the desired phosphoramidite **6** (73.5 g, 88%) as an amorphous yellow solid: *R*_*f*_ = 0.43 (PE–EtOAc, 3:1); mp 142–143 °C (melts and decomposes); ^1^H NMR (400 MHz, CDCl_3_): *δ* 7.63 (s, 2H, H-3, H-3′), 7.30 (s, 2H, H-6, H-6′), 5.150 (dd, *J* = 16.4 Hz, *J* = 6.9 Hz, 1H, ArC*H*_2_O), 5.149 (dd, *J* = 16.4 Hz, *J* = 6.9 Hz, 1H, ArC*H*_2_O), 5.061 (dd, *J* = 16.4 Hz, *J* = 6.9 Hz, 1H, ArC*H*_2_O), 5.059 (dd, *J* = 16.4 Hz, *J* = 6.9 Hz, 1H, ArC*H*_2_O), 3.87 (s, 6H, OC*H*_*3*_ × 2), 3.86 (s, 6H, OC*H*_*3*_ × 2), 3.73–3.64 (m, 2H, NC*H*(CH_3_)_2_ × 2), 1.19 (d, *J* = 6.8 Hz, 12H, NCH(C*H*_*3*_)_2_ × 2) ppm; ^13^C NMR (100 MHz, CDCl_3_): *δ* 153.9 (*C*-5, *C*-5′), 147.6 (*C*-4, *C*-4′), 138.8 (*C*-2, *C*-2′), 131.74, 131.66 (*C*-3, *C*-3′), 109.4 (*C*-1, *C*-1′), 107.9 (*C*-6, *C*-6′), 62.6 (Ar*C*H_2_O), 62.4 (Ar*C*H_2_O), 56.39 (O*C*H_3_ × 2), 56.35 (O*C*H_3_ × 2), 43.5 (N*C*H(CH_3_)_2_), 43.4 (N*C*H(CH_3_)_2_), 24.8 (NCH(*C*H_3_)_*2*_); 24.7 (NCH(*C*H_3_)_*2*_) ppm; ^31^P NMR (162 MHz, CDCl_3_): *δ* + 147.41 ppm.

##### 6-O-Bis-(4,5-dimethoxy-2-nitrobenzyloxyphosphoryl)-d-trehalose (DMNB-T6P)



Potassium carbonate (165 mg, 1.19 mmol) was added into methanol (165 ml, HPLC grade, 5.5 ml g^−1^), and the resulting suspension was stirred at room temperature for 30 min. Then, CH_2_Cl_2_ (33 ml, HPLC grade, 1.1 ml g^−1^) was added, followed by the addition of the substrate **1** (30 g, 32.62 mmol) in one portion. After stirring for 2 h at room temperature, the clear solution was quenched by acetic acid (136.7 μl, 2.39 mmol) and pyridine (193 μl, 2.39 mmol) successively. After removal of the solvent under vacuum, the crude residue was then suspended in CH_2_Cl_2_ (200 ml) and washed with saturated NaCl solution (200 ml). The organic layer was separated; the aqueous layer was extracted with CH_2_Cl_2_ (50 ml × 3); and the combined organic layers were dried over Na_2_SO_4_, filtered and concentrated and dried under high vacuum overnight, giving a mixture of **2a** and **2b** as a white foam, which was used directly in the phosphorylation. Under inert environment (argon), a mixture of the residue from above and molecule sieve (32.6 g, 4 Å MS, powder, 100 mg ml^−1^) in dry CH_2_Cl_2_ (326 ml, 10 ml mmol^−1^) was stirred for 30 min at room temperature, and then 5-phenyl-1*H*-tetrazole (10.0 g, 68.50 mmol, 2.10 eq.) was added, followed by the addition of phosphoramidite **6** (19.0 g, 34.25 mmol, 1.05 eq.) in five portions over 2.5 h. After stirring for 30 min at room temperature, the solution was cooled to −78 °C, and *meta*-chloroperbenzoic acid (8.85 g, 35.88 mmol, 1.1 eq., ~70%) was added slowly, and the resulting mixture was allowed to warm to room temperature and was stirred for 30 min. Then, the reaction was quenched by dimethyl sulfide (479 μl, 6.52 mmol, 0.2 eq.) slowly. After stirring for 30 min, the mixture was filtered, and the filtrate was concentrated under vacuum and purified by flash column chromatography (PE–EtOAc, 1:1) to give a mixture of the trimethylsilyl (TMS)-protected intermediates as a white foam. The resulting foam was dissolved in CH_2_Cl_2_ (652 ml, HPLC grade, 20 ml mmol^−1^), and trifluoroacetic acid (32.6 ml, 5%, v/v) was added. After stirring for 30 min at room temperature, the reaction solution was completely concentrated under vacuum, giving a yellow foam (around 17 g). Recrystallization: methanol (100 ml) was added, and the suspension was heated to 55 °C to facilitate a clear solution and then cooled to room temperature slowly. After repeating this ‘heating–cooling’ operation three times, yellow powder appeared. After that, it was left at 4 °C overnight, and the yellow solid was collected by filtration, giving the desired product DMNB-T6P (13.3 g, 50%) as an amorphous yellow powder that was then recrystallized to give a yellow solid: *R*_*f*_ = 0.23 (EtOAc–CH_3_OH, 2:1; or EtOAc–CH_3_OH, 3:1, plus 0.1% of formic acid (v/v)); mp 124–125 °C (melts and decomposes); [α]_D_^25^ + 63.2 (*c* 1.0, CH_3_OH); ^1^H NMR (400 MHz, CD_3_OD): *δ* 7.63 (d, *J*_*p*_ = 0.8 Hz, 2H, ArH), 7.13 (s, 2H, ArH), 5.48–5.44 (m, 4H, ArC*H*_*2*_O × 2), 5.04 (d, *J*_1,2_ = 3.7 Hz, 1H, H-1), 5.00 (d, *J*_1′,2′_ = 3.7 Hz, 1H, H-1′), 4.42–4.32 (m, 2H, H-6a, H-6b), 4.05–4.01 (m, 1H, H-5), 3.91 (s, 6H, OC*H*_*3*_ × 2), 3.88 (s, 6H, OC*H*_*3*_ × 2), 3.82–3.72 (m, 4H, H-5′, H-6′a, H-3, H-3′), 3.66 (dd, *J*_6′b,6′a_ = 12.0 Hz, *J*_6′b,5′_ = 5.3 Hz, 1H, H-6′b), 3.44 (dd, *J*_2,3_ = 8.2 Hz, *J*_2,1_ = 3.7 Hz, 1H, H-2), 3.41 (dd, *J*_2′,3′_ = 8.2 Hz, *J*_2′,1′_ = 3.7 Hz, 1H, H-2′), 3.34 (dd, *J*_4,3_ = 9.9 Hz, *J*_4,5_ = 9.0 Hz, 1H, H-4), 3.30 (t, *J*_4′,3′_ = 9.5 Hz, *J*_4′,5′_ = 9.5 Hz, 1H, H-4′H) ppm; ^13^C NMR (100 MHz, CD_3_OD) *δ* 155.2 (q*C*Ar), 150.0 (q*C*Ar), 140.8 (q*C*Ar), 140.7 (q*C*Ar), 127.73 (d, ^3^*J*_P,C_ = 6.6 Hz, q*C*Ar), 127.66 (d, ^3^*J*_P,C_ = 6.6 Hz, q*C*Ar), 111.6 (Ar*C*), 111.5 (Ar*C*), 109.3 (Ar*C*), 95.34 (*C*-1), 95.27 (*C*-1′), 74.6 (*C*-3), 74.4 (*C*-3′), 73.9 (*C*-5′), 73.15 (*C*-2), 73.06 (*C*-2′), 72.0 (d, ^3^*J*_P,C5_ = 6.4 Hz, *C*-5), 71.9 (*C*-4), 71.2 (*C*-4′), 68.8 (d, ^2^*J*_P,C6_ = 5.7 Hz, *C*-6), 67.91 (d, ^2^*J*_P,C_ = 4.4 Hz, Ar*C*H_2_O), 67.88 (d, ^2^*J*_P,C_ = 4.4 Hz, Ar*C*H_2_O), 62.6 (*C*-6′), 57.0 (O*C*H_3_), 56.8 (O*C*H_3_) ppm; high-resolution mass spectrometry (HRMS) (ESI): *m*/*z* was calculated for C_30_H_41_O_22_N_2_NaP [M+Na]^+^ 835.1781. Found: 835.1772.

### DMNB-T6P treatment

DMNB-T6P was dissolved in DMSO with Tween 20 as adjuvant (Supplementary Table 1) fresh for delivery to the crop using a backpack CO_2_ sprayer with flat fan type nozzle at a flow of 200 L per hectare, covering the whole plot.

### Field trial at CIMMYT, Mexico

Seeds were sown at the CIMMYTʼs Campo Experimental Norman E. Borlaug (CENEB) outside of Ciudad Obregon, Sonora, Mexico (27.372035, −109.924919). The soil type at the experimental station is a coarse sandy clay, mixed montmorillonitic typic caliciorthid, low in organic matter and slightly alkaline (pH 7.7)^59^. Appropriate weed disease and pest control were implemented to avoid yield limitations. Plots were fertilized with 50 kg N per hectare (urea) and 50 kg P per hectare at soil preparation, 50 kg N per hectare with the first irrigation and another 150 kg N per hectare with the second irrigation. Four high-yielding, modern, semi-dwarf, spring wheat genotypes were grown: BACANORA T 88, KAUZ*2/MNV//KAUZ, KAMBARA2 and BORLAUG100 F2014. The plants were sown on 16 December 2021 in a randomized split plot design with DMNB-T6P treatments applied to main plots and cultivars randomized to subplots. Each plot consisted of two beds with two rows, 3.5 m in length. Dose was varied by concentration; four DMNB-T6P treatments were applied in the field: the control (0 T6P), 0.5 mM, 1 mM and 2 mM DMNB-T6P in the volume per m^2^ equivalent to dose 2 adjusted for the sprayed area (Supplementary Table 1). Preparation of the T6P solution was as previously described^23^ and as for the field trials in Argentina. The DMNB-T6P solution was applied once to the canopy of the wheat crop in the late afternoon at 10 DAA.

The field trial was harvested on 31 May 2022, after reaching full maturity. Yield components were evaluated following the CIMMYT Wheat Physiology Handbook^60^. Fifty tillers were harvested at random per plot and then brought to the field station at CENEB for further processing. After harvesting the tillers, the spikes were removed from the stems and dried in an oven until reaching a dry constant weight. Seeds were then threshed and used to calculate thousand grain weight (TGW) and grain number (GN). Border plants were excluded from both the final and yield component harvests to minimize border effects between genotypes and treatments.

LEF was measured using a MultispeQ 2.0 (PhotosynQ) and the pre-programmed RIDES protocol. No significant difference was observed between treatments for ambient photosynthetic photon flux density (PPFD) at the time of measurement, indicating that differences in light intensity are not a contributing factor to differences seen between genotypes or treatments (Extended Data Fig. 8). Measurements were made in the field between 10:30 and 14:30 on the wheat flag leaf 3 d after the foliar application of the DMNB-T6P solution. In total, six plants (*n* = 6) were measured per genotype and treatment. Six plots were measured per genotype and treatment (*n* = 6). Within the plot, two plants were measured.

### Field trials in Argentina

Over four seasons (2018, 2020, 2021 and 2022), field trials were performed under rainfed conditions at the National Institute of Agricultural Research (INTA) Oliveros Research Station, Santa Fe, Argentina (32° 3′ S, 60° 51′ W), in an argiudoll soil with more than 50 years of agricultural history^61^. High-yielding commercial Argentinian spring wheat bread-making varieties were chosen with 13–15% grain protein: Buck Saeta, DM Ceibo and MS INTA 415. Buck Saeta is Group 1, suitable for industrial baking. Ceibo is Group 2, suitable for traditional baking (more than 8 h of fermentation). MS INTA is Group 3, suitable for direct baking (less than 8 h of fermentation). No tillage conditions were used following soybean as the previous crop. Dose was varied by spray volume. DMNB-T6P was applied once at 1 mM in two or three separate doses (different volumes) (doses 1–3, at 220 ml, 438 ml or 656 ml per 7-m^2^ plot; Supplementary Table 1). Application was at 10 DAA in 2018, 2020 and 2022 and at 16 DAA in 2021 (due to late delivery of DMNB-T6P), applied in the morning. Calendar timings are shown in Supplementary Table 2. Treatments were arranged in a randomized complete block design with 4–6 replications. Each experimental unit was seven rows spaced 20 cm and 7 m long. The central five rows of each plot were sprayed, giving a spray area of 7 m^2^, of which 3 m^2^ (three central rows 0.6 m × 5 m long) was harvested for grain yield. Phosphorus, sulphur and nitrogen fertilization was performed using super triple phosphate (20% P), calcium sulphate (18% S) and urea, applied at planting at a rate of 100 kg per hectare. N fertilization was estimated by summing pre-plant soil N test as nitrates at 0–60-cm depth (PPNT) plus N added as fertilizer to reach 140 kg per hectare as urea–ammonium nitrate (32% N). N rates were 130, 119, 77 and 101 for years 1, 2, 3 and 4, respectively. Soil organic matter was 2.3% in year 1, 2.5% in year 2, 2.6% in year 3 and 1.9% in year 4, and pH was 5.5, 6.1, 5.9 and 5.8 in the 4 years, respectively.

### Weather conditions during the wheat cycle

Cumulative rainfall from May (before crop planting and important for recharging the soil profile) to middle November (when physiological maturity was reached) was 544 mm, 119 mm, 290 mm and 130 mm in years 1, 2, 3 and 4, respectively (Extended Data Fig. 3). These values were 40% above, 70% below, 26% below and 67% below historical records. During the grain filling period (late October to early November), rainfall in years 1, 2, 3 and 4 averaged 125 mm, 58 mm, 74 mm and 39 mm, respectively (9% higher and 49%, 35% and 66% lower than historical records). Maximum and minimum temperature during the cycle ranged averaged from 22.0 °C to 23.7 °C and from 7.1 °C to 8.5 °C in the 4 years. During the grain filling period, maximum temperatures averaged 27.4 °C, and minimum temperatures averaged 12.2 °C. Maximum temperatures were 9% above historical values, and minimum temperatures were 6% below the historical records.

### Protein determination

Protein was determined using a NIRS DS2500 analyzer (FOSS Analytical) and fitted to 14% moisture.

### Data plotting and statistical analyses

**Extended Data Fig. 10:**
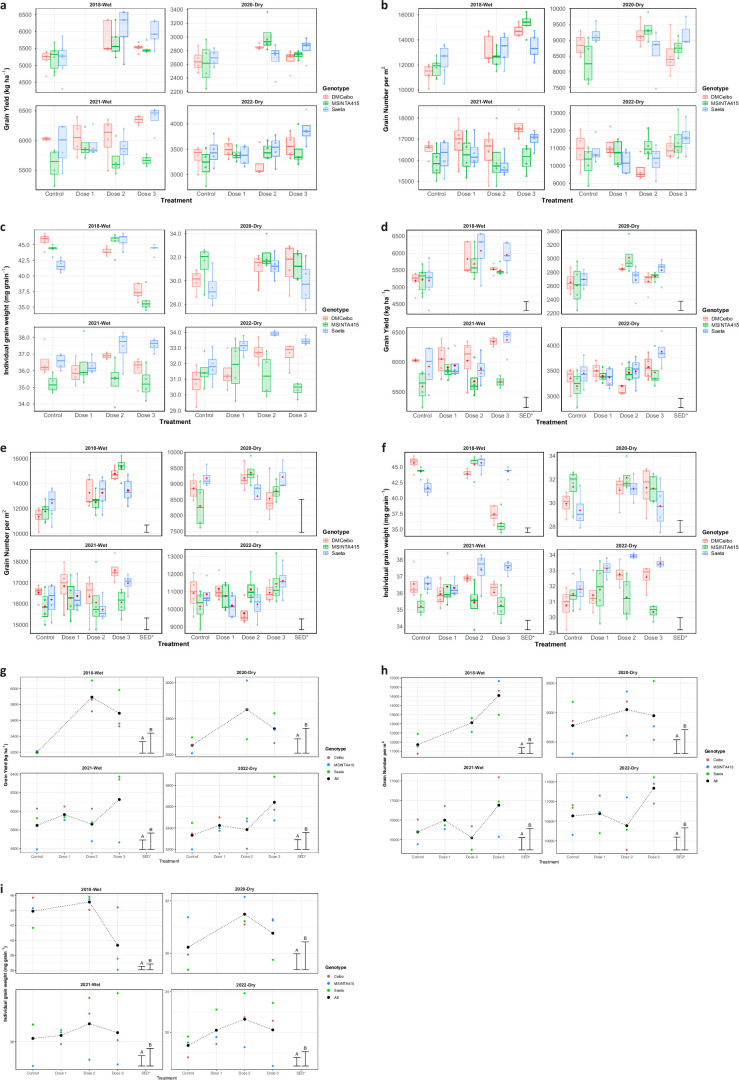
Additional data plot formats. **(a)** Varied scale boxplots of three spring wheat varieties DM Ceibo, MS INTA 415 and Saeta grown 2018–2022 in wet and dry years at two dose rates of DMNB-T6P in 2018, 2020 and three dose rates in 2021, 2022 compared to control with no DMNB-T6P. p < 0.0001, 0.065, 0.0348, 0.010 for 2018, 2020, 2021, 2022, respectively. Each data point represents an individual field plot. Median is shown as box line. **(b)** Varied scale boxplots of grain number per m2 in response to DMNB-T6P spray over four seasons. Three spring wheat varieties DM Ceibo, MS INTA 415 and Saeta grown 2018–2022 at two dose rates of DMNB-T6P in 2018, 2020 and three dose rates of DMNB-T6P in 2021, 2022 compared to control with no DMNB-T6P. Each data point represents an individual plot. Median is shown as box line. **(c)** Variable scale boxplots Individual grain weight (mg) in response to DMNB-T6P spray over four seasons. Three spring wheat varieties DM Ceibo, MS INTA 415 and Saeta grown 2018–2022 at two dose rates of DMNB-T6P in 2018, 2020 and three dose rates of DMNB-T6P in 2021, 2022 compared to control with no DMNB-T6P. Each data point represents an individual plot. Median is shown as box line. **(d)** Variable scale boxplots as equivalent plot to A, also showing mean (red diamond) and the Standard Error of Difference (SED) for comparisons between pairs of means for combinations of genotype and treatment. **(e)** Variable scale boxplots as equivalent plot to B, also showing mean (red diamond) and SED for comparisons between pairs of means for combinations of genotype and treatment. **(f)** Variable scale boxplots as equivalent plot to C, also showing mean (red diamond) and SED for comparisons between pairs of means for combinations of genotype and treatment. **(g)** Mean grain yield (kg ha^−1^) from four field experiments over four separate occasions (years), showing overall means for each T6P treatment (black dots and lines) and means for each genotype for each T6P treatment (red dot – Ceibo, blue dot – INTA415, green dot – Saeta). SEDs shown for comparisons between pairs of overall T6P treatment means (SED type A) and for comparisons between pairs of means for combinations of genotype and T6P treatment (SED type B), based on 34, 24, 33, and 33 degrees of freedom for the 2018, 2020, 2021 and 2021 experiments, respectively. For all experiments except 2018 the replication levels are the same for all treatments, but for the 2018 experiment the T6P plots were only measured for 5 of the 6 replicate blocks – the SED A shown (145.6) is for comparing the means for the two T6P doses, with a smaller SED (139.4) for comparing the mean for the Control with that for either of the T6P doses; the SED B shown (252.1) is for comparing means for two treatments with this lower replication level (that is comparisons between genotypes at either T6P dose, or between doses for each genotype), with smaller SEDs for comparing the mean for the Control treatment with that for either dose for each genotype (241.4) and for comparing means for genotypes for the Control treatment (230.2). **(h)** Mean grain number per m^2^ from four field experiments over four separate occasions (years), showing overall means for each T6P treatment (black dots and lines) and means for each genotype for each T6P treatment (red dot – Ceibo, blue dot – INTA415, green dot – Saeta). SEDs shown for comparisons between pairs of overall T6P treatment means (SED type A) and for comparisons between pairs of means for combinations of genotype and T6P treatment (SED type B), based on 34, 24, 33, and 33 degrees of freedom for the 2018, 2020, 2021 and 2021 experiments, respectively. For all experiments except 2018 the replication levels are the same for all treatments, but for the 2018 experiment the T6P plots were only measured for 5 of the 6 replicate blocks – the SED A shown (333.7) is for comparing the means for the two T6P doses, with a smaller SED (319.5) for comparing the mean for the Control with that for either of the T6P doses; the SED B shown (578.0) is for comparing means for two treatments with this lower replication level (that is comparisons between genotypes at either T6P dose, or between doses for each genotype), with smaller SEDs for comparing the mean for the Control treatment with that for either dose for each genotype (553.4) and for comparing means for genotypes for the Control treatment (527.6). **(i)** Mean individual grain weight (mg per grain) from four field experiments over four separate occasions (years), showing overall means for each T6P treatment (black dots and lines) and means for each genotype for each T6P treatment (red dot – Ceibo, blue dot – INTA415, green dot – Saeta). SEDs shown for comparisons between pairs of overall T6P treatment means (SED type A) and for comparisons between pairs of means for combinations of genotype and T6P treatment (SED type B), based on 34, 24, 33, and 33 degrees of freedom for the 2018, 2020, 2021 and 2021 experiments, respectively. For all experiments except 2018 the replication levels are the same for all treatments, but for the 2018 experiment the T6P plots were only measured for 5 of the 6 replicate blocks – the SED A shown (0.441) is for comparing the means for the two T6P doses, with a smaller SED (0.422) for comparing the mean for the Control with that for either of the T6P doses; the SED B shown (0.764) is for comparing means for two treatments with this lower replication (that is comparisons between genotypes at either T6P dose, or between doses for each genotype), with smaller SEDs for comparing the mean for the Control treatment with that for either dose for each genotype (0.731) and for comparing means for genotypes for the Control treatment (0.697). Box plots a-f range from the first (Q1) to the third quartile (Q3) of the distribution and contain the 25th to 75th percentiles of dataset, respectively, representing the interquartile range (IQR). The centre line inside each box represents the median value (50th percentile). Whiskers extending below Q1 and above Q3 denote minimum and maximum values of the dataset within 1.5 x IQR from the 25th and 75th percentiles, respectively.

Data are plotted as box plots (Figs. 2–4), which plot the data with medians but not the statistical tests. Statistical analysis of each Argentinian field trial was performed using a two-way factorial ANOVA accounting for the randomized complete block layout in R version 4.2.1. Additionally, a combined analysis over all 4 years was performed using a mixed model framework fitted using REML (Supplementary Table 3). The model consisted of variance components for both block and the blockplot residual separately for each year. Approximate (sequential) *F* statistics were calculated using Kenward–Roger degrees of freedom. Additionally, standard errors of the difference (SEDs) of the means are plotted as supplementary data (Extended Data Fig. 10). SEDs are shown for comparisons between pairs of overall T6P treatment means and for comparisons between pairs of means for combinations of genotype and T6P treatment based on 34, 24, 33 and 33 degrees of freedom for 2018, 2020, 2021 and 2022 experiments, respectively. Pairwise *t*-tests were conducted for Fig. 5ciii,d. Analysis of the Mexico field trial was conducted using multi-strata ANOVA to account for the split plot design. Models were fitted in Genstat 22nd edition. We avoid strict thresholding of *P* values and use of terms ‘significant’ and ‘non-significant’, as biological significance is best understood through examination of statistical tests and *P* values as a whole over the trialing period incorporating ANOVA analyses (Figs. 2–4) and combined analysis (Supplementary Table 3). We include *P* values lower than *P* < 0.1 and do not consider values higher than this. Although, of note, for acceptance as a new biostimulant in the European Union under regulations 2019/1009, such as DMNB-T6P, *P* < 0.15 values are required (European Document CEN/TS 17700-1:2022, ‘Plant Biostimulants - Claims - Part1: General Principles’ Annex A ‘P-value choice and impact on the results quality’).

### Transcriptome analyses

Whole ears were sprayed 10 DAA with 1 mM DMNB-T6P on Cadenza wheat grown in a controlled environment as in ref. ^23^. The middle-third of each ear was frozen in liquid nitrogen and stored at −80 C. Whole grain tissue was ground to a fine powder under liquid nitrogen, and total RNA was extracted using the TRIzol method for four independent biological replicates per condition at time 0, 4 h and 24 h after treatment with DMNB-T6P. After RNA integrity analysis and quantitation (Agilent, Bioanalyzer), poly(A)-enriched cDNA libraries were generated and sequenced on an Illumina NovaSeq 6000 sequencing platform generating 30–50 million 150-bp paired-end reads per sample. Low-quality reads and adaptor sequences were removed with Trimmomatic (trimmomatic-0.39.jar PE ILLUMINACLIP:TruSeq3-PE.fa:2:30:10:2:True TRAILING:30 MINLEN:40)^62^. The reads were aligned to the wheat reference genome (*Triticum aestivum* iwgsc_refseqv2.1 (ref. ^63^)) using HISAT2/2.2.1-foss-2019b with default parameters^64^ and converted to BAM format with SAMtools^65^. Gene or transcript abundance was quantified using featureCounts^66^ with the High Confidence iwgsc_refseqv2.1 annotation (counting only primary alignments of read pairs with a quality cutoff of 10). The RNA-seq data were deposited under BioProject in the National Center for Biotechnology Information (NCBI) Sequence Read Archive (SRA) (Supplementary Table 5)^67^. Raw counts were normalized using the trimmed mean of M-values method by DESeq2 (ref. ^67^). Differentially expressed genes (DEGs) were identified based on DESeq2 3.15 with adjusted *P* value (*P*_adj_) < 0.05 and |log_2_ fold change (FC)| > 1 as selection criteria. Further statistical analyses and visualizations were conducted in R, and plots and heatmaps were created using the ggplot2 3.4.0, ComplexHeatmap 2.14.0 and tidyheatmap 1.10.0 packages in R^68–70^.

### Microscopy

Whole grains were fixed in 4% paraformaldehyde with 2.5% glutaraldehyde, dehydrated in an ethanol series and embedded in LR White resin (TAAB Laboratories Equipment, Ltd.). Transverse sections of the medial region were imaged after staining with toluidine blue. All samples were imaged with a ×10 objective using an Axio Imager.Z2 (Zeiss). Sieve tube areas in the vascular bundle were manually traced and quantified with ImageJ^71^.

### Gas exchange

Leaf gas exchange measurements of Cadenza wheat were made with a portable infrared open gas exchange system (LI-COR, LI-6400XT) under the following growing conditions: ambient CO_2_ (400 µl l–1), leaf temperature 22 °C, PPFD 500 µmol m^−2^ s^−1^ and relative air humidity 65 ± 5% with an air flow rate of 200 µmol s^−1^. The middle region of each flag leaf reached a steady state of CO_2_ uptake in the leaf chamber before measurements were taken. Data are of four measurements taken at 10 DAA (before treatment), 11 DAA, 12 DAA, 15 DAA and 20 DAA from four separate plants treated with 1 mM DMNB-T6P applied to the spike at 10 DAA after growing under previously described conditions^23^.

### Treatment of sorghum with DMNB-T6P in controlled environment

Sweet sorghum seeds were grown in 30-cm pots containing Rothamsted compost^23^ under 28 °C/22 °C, 12-h day/night cycles, 500 µmol m^−2^ s^−1^ quanta and 60% relative humidity. Regular watering was continued throughout the experiment except for drought stress treatments where watering was reduced to 60% of pot weight at anthesis and maintained at that level of drought until harvest. Eight milliliters per spike of 2 mM DMNB-T6P or control without DMNB-T6P with spray composition as for Argentinian experiments (Supplementary Table 2) was applied to spike only at 7 DAA and 14 DAA. Spikes were harvested at maturity; grain yield was measured; and significance was calculated by Studentʼs *t*-test. Each treatment contained six biological replicates.

### Treatment of barley with DMNB-T6P in controlled environment

Spring barley seeds were grown in 21-cm pots containing Rothamsted compost^23^ under 22 °C/18 °C, 16-h day/night cycles, 500 µmol m^−2^ s^−1^ quanta and 60% relative humidity. Regular watering was continued throughout the experiment except for drought stress treatments where watering was reduced at anthesis to 60% of pot weight and maintained at that level of drought until harvest. Twenty milliliters per plant of 2 mM DMNB-T6P or control without DMNB-T6P with spray composition as for Argentinian experiments (Supplementary Table 2) was applied to the upper canopy, including spikes, at 6 DAA and 11 DAA. Spikes were harvested at maturity; grain yield was measured; and significance was calculated by Studentʼs *t*-test. Each treatment contained six biological replicates.

### Reporting summary

Further information on research design is available in the Nature Portfolio Reporting Summary linked to this article.

## Online content

Any methods, additional references, Nature Portfolio reporting summaries, source data, extended data, supplementary information, acknowledgements, peer review information; details of author contributions and competing interests; and statements of data and code availability are available at 10.1038/s41587-025-02611-1.

## Supplementary information

Supplementary Information Supplementary Tables 1–6 and Supplementary Chemical Data

Reporting Summary

Supplementary Data 1 Confidence intervals for Figs. 2–4

## Data Availability

RNA-seq reads were deposited to the NCBI SRA under BioProject ID PRJNA1007614 (ref. ^72^). RNA-seq normalized counts were deposited to a Zenodo repository: 10.5281/zenodo.8269041 (ref. ^73^); field trial data from both sites and all photosynthesis data were deposited to a Zenodo repository: 10.5281/zenodo.14882353 (ref. ^74^). Bioimaging file data are available upon reasonable request due to file size limitations. All other data are presented in the paper or the supplementary materials. Source data are provided with this paper.
