# Supplementary material for: Membrane-permeable trehalose 6-phosphate precursor spray increases wheat yields in field trials
Source: Nat Biotechnol. 2025 Apr 29;44(2):316–25. doi: 10.1038/s41587-025-02611-1 (PMC12909131; doi:10.1038/s41587-025-02611-1)

nature biotechnology

Supplementary information

https://doi.org/10.1038/s41587-025-02611-1

Membrane-permeable trehalose 6-phosphate precursor spray increases wheat yields in field trials

In the format provided by the authors and unedited

SUPPLEMENTARY INFORMATION

Supplementary Tables

Supplementary Chemical Data

SUPPLEMENTARY TABLES

Table S1. Spraying treatments of DMNB-T6P spray used in Argentina and Mexico. In Argentina three volumes (dose rates) of 1 mM DMNB-T6P were used in 2021 and 2022; two volumes in 2018 and 2020, compared to control. In Mexico, dose 2 volume was used with 0.5, 1 or 2 mM DMNB-T6P.

Per 7 m^2 plot	DMNB-T6P per plot (g) for 1 mM final concentration	DMSO per plot (ml)	Water per plot (ml)	Tween 20 per per plot (ml)
Dose 1 (year 3, 4 only) Total volume 220 ml	0.167	3.60	216	0.22
Dose 2 (year 1-4) Total volume 438 ml	0.332	7.3	430	0.44
Dose 3 (year 1-4) Total volume 656 ml	0.50	10.9	644	0.66
Control (year 1-4) Total volume 547 ml	0	9.1	538	0.54

Table S2. Timings of crop development and applications of DMNB-T6P in Argentina. Dates of planting, emergence, anthesis, application of DMNB-T6P and crop harvest.

	Planting	Emergence	Anthesis	Application	Harvest
2018	June 28	July 14	October 12	October 23	December 6
2020	June 30	July 15	October 23	November 3	December 1
2021	June 25	July 12	October 14	October 29	November 20
2022	June 30	July 16	October 11	October 21	November 25

Table S3. Combined analysis over all 4 years using a mixed model framework fitted using restricted maximum likelihood (REML). The model consisted of variance components for both block and the block.plot residual separately for each year. Approximate (sequential) F statistics were calculated using the Kenward-Roger degrees of freedom. Models were fitted in Genstat 22nd edition.

	Fixed term	ndf	Fstatistic	ddf	Fpr
Grain yield	Genotype	2	5.58	103.7	0.005
	Treatment	3	8.86	94.5	<0.001
	Year.Genotype	6	3.55	81.6	0.004
	Year.Treatment	7	3.75	85	0.001
	Genotype.Treatment	6	2.86	96.9	0.013
	Year.Genotype.Treatment	14	0.74	92.6	0.732
Individual grain weight	Genotype	2	24.49	104.7	<0.001
	Treatment	3	20.38	93.6	<0.001
	Year.Genotype	6	4.09	75.7	0.001
	Year.Treatment	7	21.37	80.5	<0.001
	Genotype.Treatment	6	12.34	94.1	<0.001
	Year.Genotype.Treatment	14	8.1	88.6	<0.001
Grain number per m^2	Genotype	2	0.67	109.7	0.513
	Treatment	3	13.34	100.3	<0.001
	Year.Genotype	6	1.46	82.5	0.203
	Year.Treatment	7	6.62	84.5	<0.001
	Genotype.Treatment	6	1.85	103.7	0.097
	Year.Genotype.Treatment	14	2.45	92.1	0.005

Table S4. Yield of sorghum and barley treated with 2 mM DMNB-T6P in controlled environment. Plants grown under full hydration until anthesis when watering was decreased to maintain pot weight at 60% of fully hydrated weight. Control plants maintained at full hydration. DMNB-T6P applied to spikes only in sorghum and whole canopy of barley. Yield of spikes of six biological replicates per treatment. p value from Student's t-test.

	Water regime	Treatment	Spike weight (g)	% yield increase	SEM	*p* value
Sorghum	Well-watered	control	49.25		0.424	
		DMNB-T6P	56.72	+15.2	0.779	1.47×10^{-9}
	Drought	control	31.73		0.766	
		DMNB-T6P	39.45	+24.3	0.591	1.09×10^{-8}
Barley	Well-watered	control	57.05		1.310	
		DMNB-T6P	68.08	+19.3	1.221	0.0001
	Drought	control	53.65		0.934	
		DMNB-T6P	59.43	+10.8	0.622	0.0004

Table S5. Summary of RNA-seq metrics from raw reads to counted features. Paired end raw read per sample, percentage of reads discarded by trimmomatic due to quality threshold and adapter content, overall percentage of aligned reads by HISAT2 against *Triticum aestivum* IWGSC_v2.1 genome, number of features counted at gene level by featureCounts.

sample	read_ n_raw	trimmomatic_dropped pct	hisat2_overall_ alignment_pct	FeatureCounts_assigned_ alignments
GrNT4h1	3.42E+07	0.02	93.69	2.14E+07
GrNT4h2	3.61E+07	0.02	93.71	2.00E+07
GrNT4h3	3.61E+07	0.02	94.14	2.33E+07
GrNT4h4	3.09E+07	0.02	93.64	1.88E+07
GrT6P4h1	5.24E+07	0.02	94.95	2.66E+07
GrT6P4h2	3.01E+07	0.02	93.43	1.97E+07
GrT6P4h3	3.78E+07	0.02	93.17	2.24E+07
GrT6P4h4	3.33E+07	0.02	92.41	2.03E+07
GrNT24h1	5.00E+07	0.03	93.12	3.32E+07
GrNT24h2	3.71E+07	0.02	93.46	2.47E+07
GrNT24h4	5.59E+07	0.03	93.16	3.62E+07
GrT6P24h1	3.48E+07	0.02	93.83	2.19E+07
GrT6P24h2	3.70E+07	0.02	94.27	2.10E+07
GrT6P24h3	2.98E+07	0.02	93.29	1.96E+07
GrT6P24h4	3.98E+07	0.02	94.00	2.56E+07

Table S6. DESeq2 normalised counts. TPM calculated from DESeq2 normalised counts and exonic length extracted from IWGSC_v2.1 annotation.

organ	treat	time	time_num_h	cond	normcount >= 1	norm TPM >= 0.5
Grain	Mock	10daa	0	GM000	69211	51184
Grain	Mock	4h	4	GM004	69415	50570
Grain	T6P	4h	4	GT004	69383	50813
Grain	T6P	11daa	24	GT024	68680	50661

SUPPLEMENTARY CHEMICAL DATA

^{1}H NMR of **DMNB-T6P** (400 MHz, CD_3OD)

^{13}C NMR of **DMNB-T6P** (400 MHz, CD_3OD)

^{31}P NMR of **DMNB-T6P** (162 MHz, CD_3OD)

HRMS (ESI) of **DMNB-T6P**

HPLC analysis of **DMNB-T6P**

Elemental analysis of **DMNB-T6P**

X-ray powder diffraction of **DMNB-T6P**

Stability investigation of **DMNB-T6P**

^{1}H NMR of compound **1** (400 MHz, $CDCl_3$)

^{1}H NMR of compound **2a** (400 MHz, $CDCl_3$)

^{1}H NMR of compound **2b** (400 MHz, $CDCl_3$)

^{1}H NMR of compound **3** (400 MHz, $CDCl_3$)

^{1}H NMR of compound **4** (400 MHz, $CDCl_3$)

^{1}H NMR of compound **6** (400 MHz, $CDCl_3$)

^{13}C NMR of compound **6** (100 MHz, $CDCl_3$)

^{31}P NMR of compound **6** (162 MHz, $CDCl_3$)

^{1}H NMR of **DMNB-T6P** (400 MHz, CD_3OD)

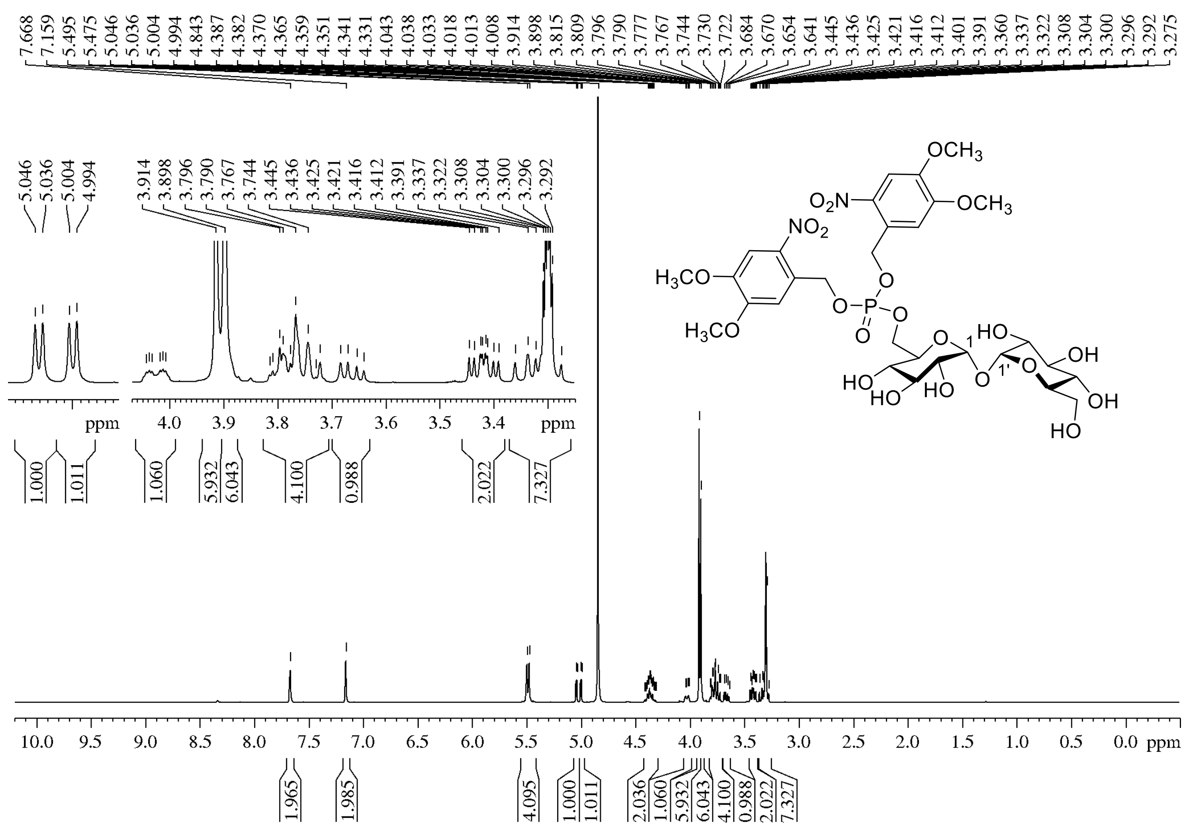

^{13}C NMR of **DMNB-T6P** (100 MHz, CD_3OD)

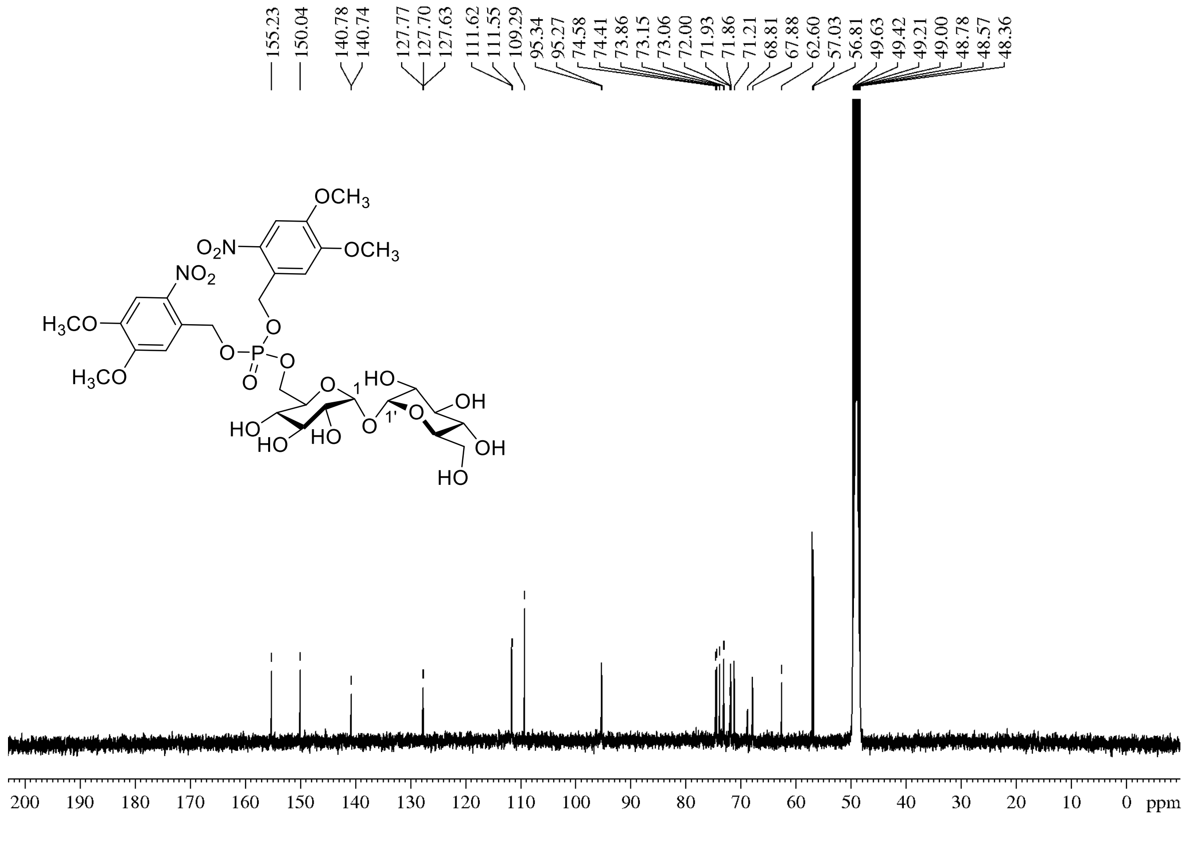

^{31}P NMR of **DMNB-T6P** (162 MHz, CD_3OD)

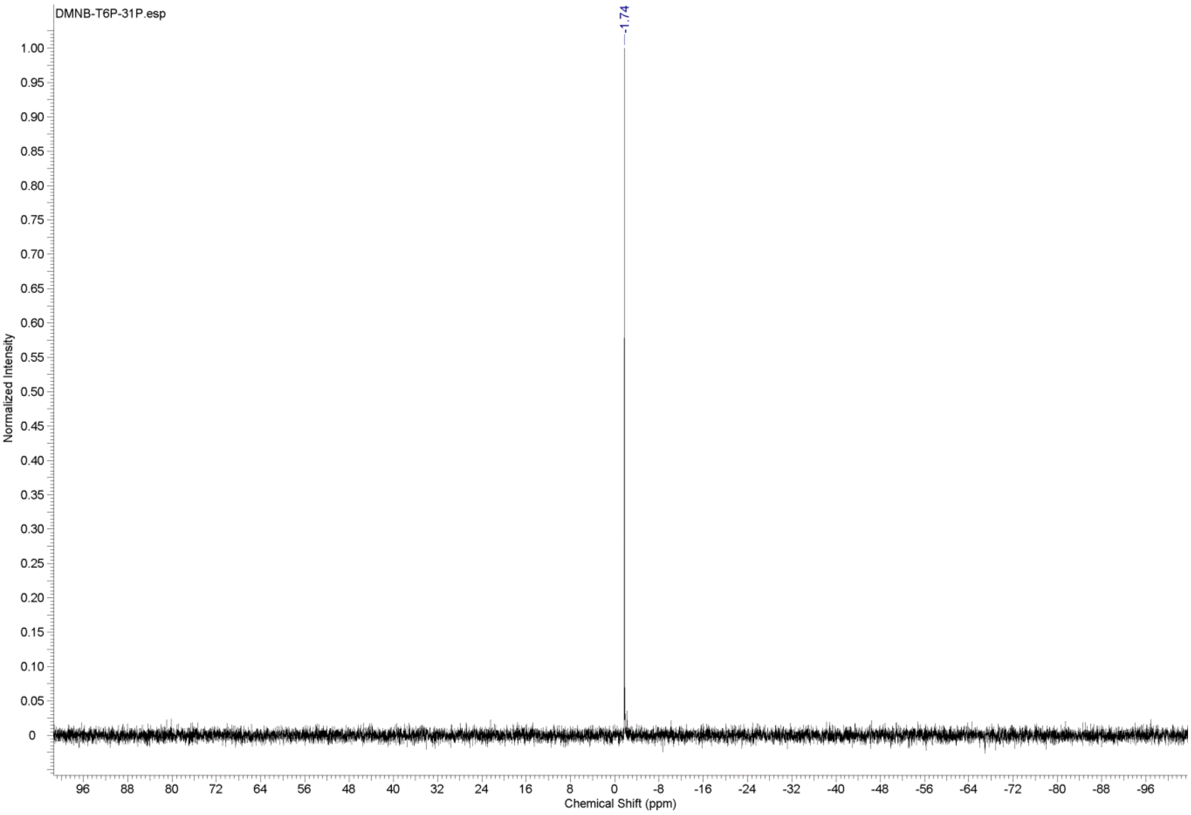

2D ^{1}H-^{1}H COSY of **DMNB-T6P** (CD_3OD)

HRMS (ESI) of **DMNB-T6P**

X:\data\May 18\ESI69429.raw 03/05/2018 3:35 pm

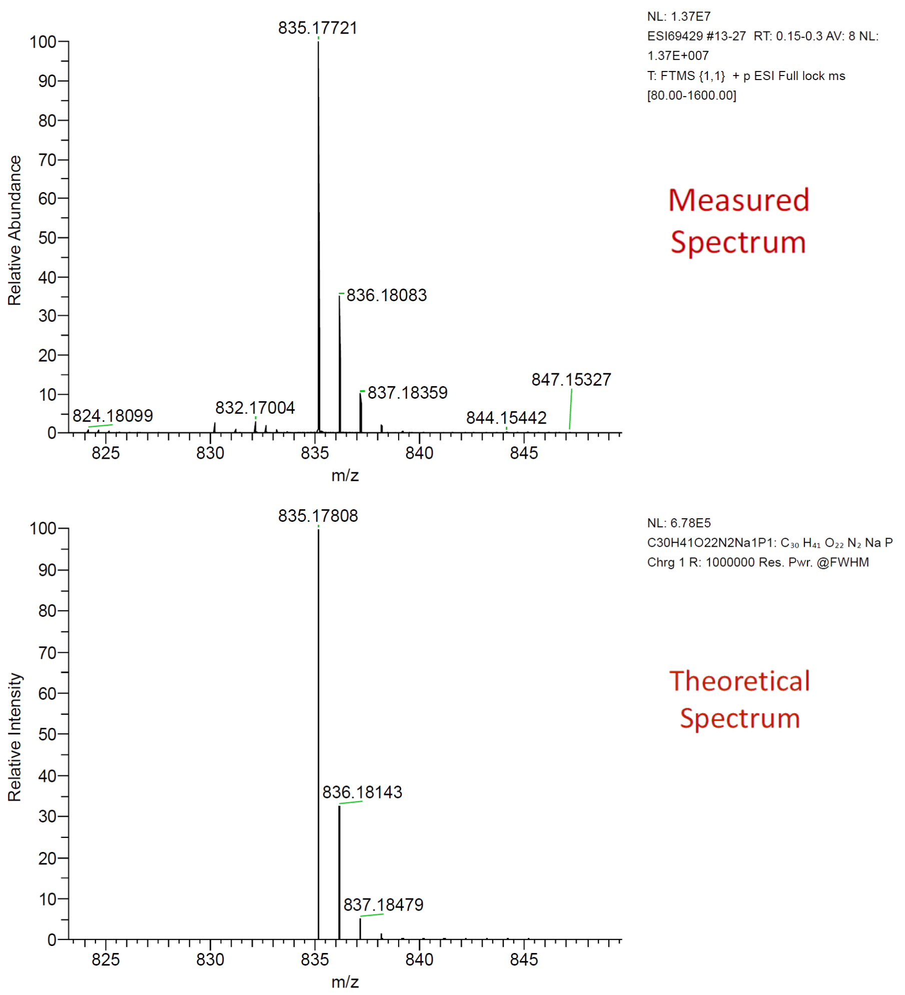

m/z	Formula	RDB	Delta ppm	Theo. Mass
835.17719	$C_{30}H_{41}O_{22}N_2{}^{23}NaP$	11.5	-1.07	835.17808

HPLC analysis of **DMNB-T6P**

Analysis Report

SHIMADZU LabSolutions

<Sample Information>

Sample Name	: DMNB T6P
Sample ID	: DMNB T6P
Data Filename	: DMNB T6P 1 Mm.lcd
Method Filename	: takuya-analysis-e70-lcmscolumn.lcm
Batch Filename	: DMNB-T6P.lcb
Vial #	: 1-79
Injection Volume	: 20 uL

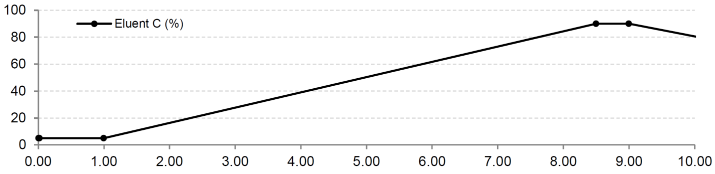

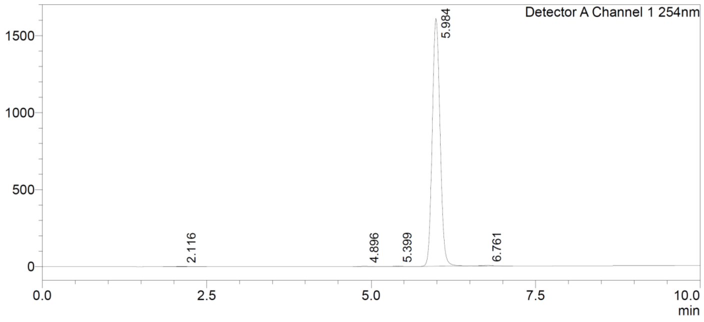

Sample: 1 mM (H_2O-CH_3OH, 1:1, v/v)

Column: Nova-Pak® C_{18}, 3.9*150 mm Column

Part No. WAT086344

Solvent Systems (Gradient):

Eluent A: H_2O (0.1% FA)

Eluent C: CH_3CN (0.1% FA)

Total Flow: 1.00 ml/min

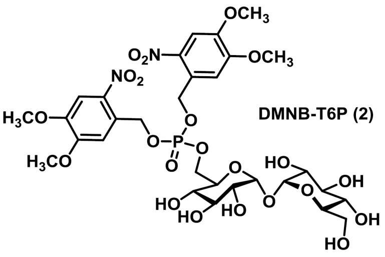

DMNB-T6P (2)

Peak#	Ret. Time	Area	Height	Area%
1	2.116	19412	1522	0.145
2	4.896	14812	1907	0.111
3	5.399	17304	2021	0.129
4	5.984	13273939	1608742	99.267
5	6.761	46535	4404	0.348
Total		13372002	1618596	100.000

Elemental analysis of DMNB-T6P

Elemental Microanalysis

Microanalysis Report

Your Reference: CHAO-DMNB-T6P

UKAS TESTING 4260

Report R16937 | **Sample S16748**

Xiaochao Xue
Oxford University

Administrator
Chemistry Research Laboratory
Mansfield Road
Oxford
OX1 3TA

Purchase Order	DW2098081
Telephone	
Fax	
Mobile	
Email	xiaochao.xue@chem.ox.ac.uk
ccEmail	lab.elemental@gmail.com

Customer Expected Values

C	44.34
H	5.09
N	3.45
O	43.31
S	

Microanalysis Results %			U +/- %
C	43.82	43.07	0.210 %
H	5.30	5.18	0.094 %
N	3.53	3.45	0.037 %
O	39.19	37.38	0.235 %
S	N/A	N/A	N/A

Average Results Chart

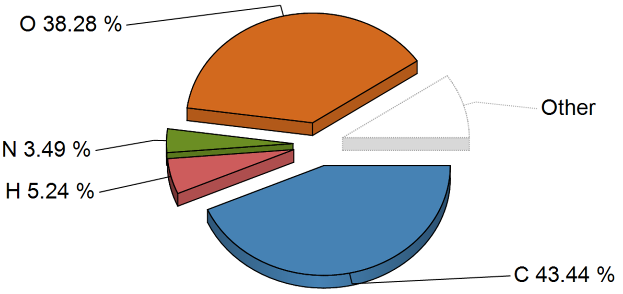

CHNS analyses performed using Dumas combustion method, O analyses performed using Unterzaucher pyrolysis method
Uncertainty values apply to individual results

Sample description and analyst comments, including any deviations from the standard method

CREAM POWDER

Analysis Date 28 November 2018

The results above apply only to the sample as received and stated in this report. This report may not be reproduced other than in full without prior written approval of the issuing laboratory

Andrew Colton (Analyst)

Elemental Microanalysis Ltd, Hameldown Road, Okehampton Business Park, Exeter Road, Okehampton, Devon
EX20 1UB Tel (01837) 54446 Fax(01837) 54544
Email: info@microanalysis.co.uk Web: www.microanalysis.co.uk

Page 1 of 1

X-ray powder diffraction of DMNB-T6P

Peak List

d-spacing [Å]	Area[cts*°2θ]	Pos.[°2θ]	Height [cts]	FWHMLeft[°2θ]	Rel. Int. [%]
20.32961	103.84	5.0473	685.78	0.1535	1.61
11.30390	1357.58	9.0840	10758.52	0.1279	25.33
8.11884	1525.26	12.6602	15109.18	0.1023	35.57
7.25007	207.31	14.1846	1369.07	0.1535	3.22
6.75455	7503.89	15.2312	42476.15	0.1791	100.00
6.39347	168.94	16.0969	1115.67	0.1535	2.63
6.04720	404.37	17.0253	3204.58	0.1279	7.54
5.81619	25.55	17.7069	253.12	0.1023	0.60
5.62431	91.31	18.3160	904.48	0.1023	2.13
5.46641	41.27	18.8499	272.53	0.1535	0.64
5.33931	572.20	19.3028	3778.81	0.1535	8.90
5.05454	1171.09	20.4016	9280.59	0.1279	21.85
4.94032	86.27	20.8786	854.60	0.1023	2.01
4.73397	796.03	21.7995	7885.45	0.1023	18.56
4.69033	464.08	22.0049	4597.19	0.1023	10.82
4.57544	167.23	22.5646	1325.24	0.1279	3.12
4.40510	952.27	23.4493	7546.54	0.1279	17.77
4.19460	41.83	24.6259	348.54	0.0900	0.82
4.11986	131.35	25.0984	1734.87	0.0768	4.08
4.04375	1666.99	25.5787	13210.48	0.1279	31.10
3.91341	252.40	26.4458	2000.23	0.1279	4.71
3.82881	142.90	27.0411	808.91	0.1791	1.90
3.74919	294.99	27.6266	1669.80	0.1791	3.93
3.61886	256.08	28.6424	2029.35	0.1279	4.78
3.59829	228.88	28.8097	2267.30	0.1023	5.34
3.54460	1077.64	29.2557	5337.53	0.2047	12.57
3.48416	378.23	29.7749	3746.70	0.1023	8.82
3.46272	486.03	29.9635	3851.66	0.1279	9.07
3.42774	351.71	30.2766	2322.71	0.1535	5.47
3.36291	212.94	30.8746	1406.23	0.1535	3.31
3.33459	398.57	31.1435	2632.13	0.1535	6.20
3.27325	771.98	31.7424	3398.78	0.2303	8.00
3.15287	281.06	32.9883	1392.09	0.2047	3.28
3.08391	287.80	33.7478	2850.91	0.1023	6.71
3.06666	268.55	33.9433	2128.21	0.1279	5.01
3.01053	348.45	34.5960	1972.43	0.1791	4.64
2.94181	40.46	35.4305	267.19	0.1535	0.63
2.88289	280.80	36.1794	2225.31	0.1279	5.24
2.80181	78.31	37.2644	775.77	0.1023	1.83
2.69711	117.52	38.7675	931.32	0.1279	2.19
2.65094	118.77	39.4704	784.38	0.1535	1.85
2.58753	230.96	40.4794	762.63	0.3070	1.80
2.53148	73.30	41.4164	414.94	0.1791	0.98
2.50904	87.77	41.8041	579.65	0.1535	1.36
2.45226	82.26	42.8190	407.42	0.2047	0.96
2.42774	128.46	43.2732	1272.48	0.1023	3.00

2.36232	210.66	44.5349	1391.18	0.1535	3.28
2.27449	35.09	46.3526	139.03	0.2558	0.33
2.25266	58.81	46.8286	388.35	0.1535	0.91
2.22651	48.45	47.4120	319.94	0.1535	0.75
2.21132	52.17	47.7577	516.78	0.1023	1.22
2.19281	167.28	48.1864	552.35	0.3070	1.30
2.15568	118.22	49.0704	334.61	0.3582	0.79
2.11980	65.74	49.9576	651.23	0.1023	1.53
2.05415	40.51	51.6696	267.54	0.1535	0.63
2.01655	92.64	52.7065	734.15	0.1279	1.73
1.99404	48.04	53.3481	317.25	0.1535	0.75
1.96964	37.18	54.0625	147.34	0.2558	0.35
1.93172	41.25	55.2130	326.91	0.1279	0.77
1.87926	22.81	56.8922	225.96	0.1023	0.53
1.85351	130.87	57.7566	432.13	0.3070	1.02
1.79201	25.30	59.9370	83.54	0.3070	0.20
1.76842	61.34	60.8206	303.80	0.2047	0.72
1.68676	17.44	64.1046	57.59	0.3070	0.14
1.63451	26.37	66.4129	174.14	0.1535	0.41
1.57935	30.88	69.0529	101.97	0.3070	0.24
1.43475	21.02	77.2044	26.02	0.8187	0.06

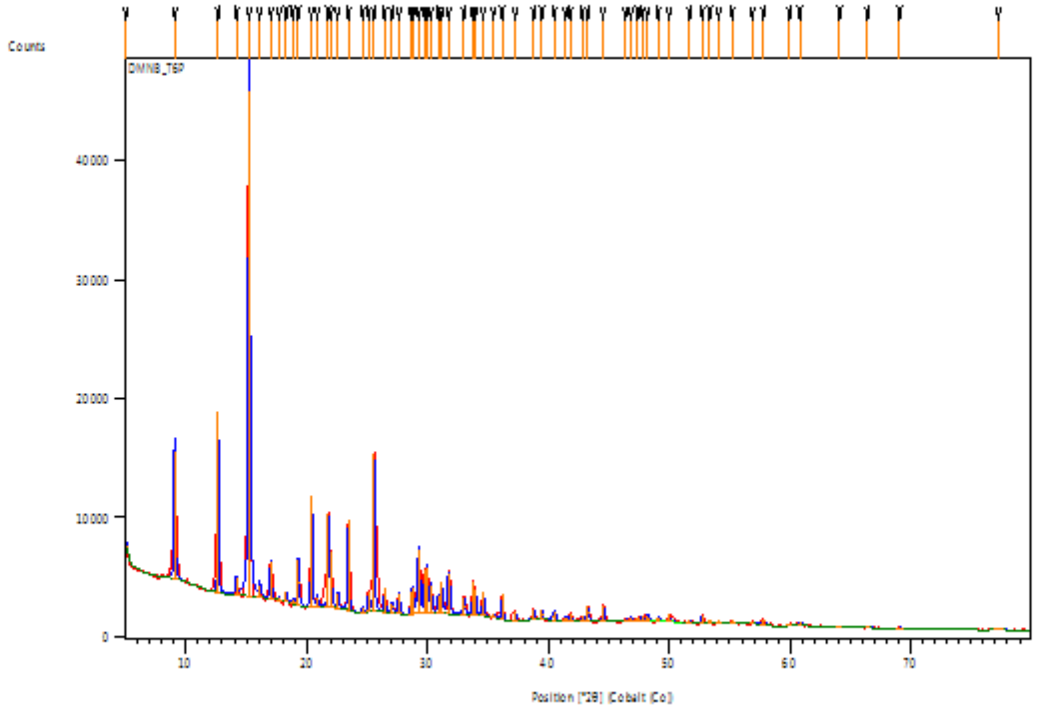

Dataset Name: DMNB_T6P

File name: C:\XRD Data\Tosca group\Kat_Data\DMNB_T6P.xrdml

Comment: Configuration=Reflection-transmission Spinner, Owner=User-1, Creation date=6/1/2015 7:12:58 PM

Goniometer=Theta/Theta; Minimum step size 2Theta:0.0001; Minimum step size Omega:0.0001

	Sample stage=Reflection-transmission spinner; Minimum step size Phi:0.1
	Diffractometer system=EMPYREAN
	Measurement program=C:\XRD Data\Programs\Nick_absolute_scan_F845.xrdmp,
	Identifier={7FA123A4-A50E-41A0-8092-9E7F33A0AACA}
	Batch program=C:\XRD Data\Programs\Tosca Group\Nick\Nick_batch_scan_2.xrdmp,
	Identifier={B3DE5272-485B-4465-B77A-8D5D0BA4FC3D}
	Ref Trans spinner
Measurement Start Date/Time:	12/5/2018 3:05:29 PM
Operator:	User
Raw Data Origin:	XRD measurement (*.XRDML)
Scan Axis:	Gonio
Start Position [°2θ]:	5.0129
End Position [°2θ]:	79.9709
Step Size [°2θ]:	0.0260
Scan Step Time [s]:	96.3900
Scan Type:	Continuous
PSD Mode:	Scanning
PSD Length [°2θ]:	3.35
Offset [°2θ]:	0.0000
Divergence Slit Type:	Fixed
Divergence Slit Size [°]:	0.5000
Specimen Length [mm]:	10.00
Measurement Temperature [°C]:	25.00
Anode Material:	Co
K-Alpha1 [Å]:	1.78901
K-Alpha2 [Å]:	1.79290
K-Beta [Å]:	1.62083
K-A2 / K-A1 Ratio:	0.50000
Generator Settings:	40 mA, 40 kV
Diffractometer Type:	0000000011167697
Diffractometer Number:	0
Goniometer Radius [mm]:	240.00
Dist. Focus-Diverg. Slit [mm]:	100.00
Incident Beam Monochromator:	No
Spinning:	Yes

^{1}H NMR of compound **1** (400 MHz, $CDCl_3$)

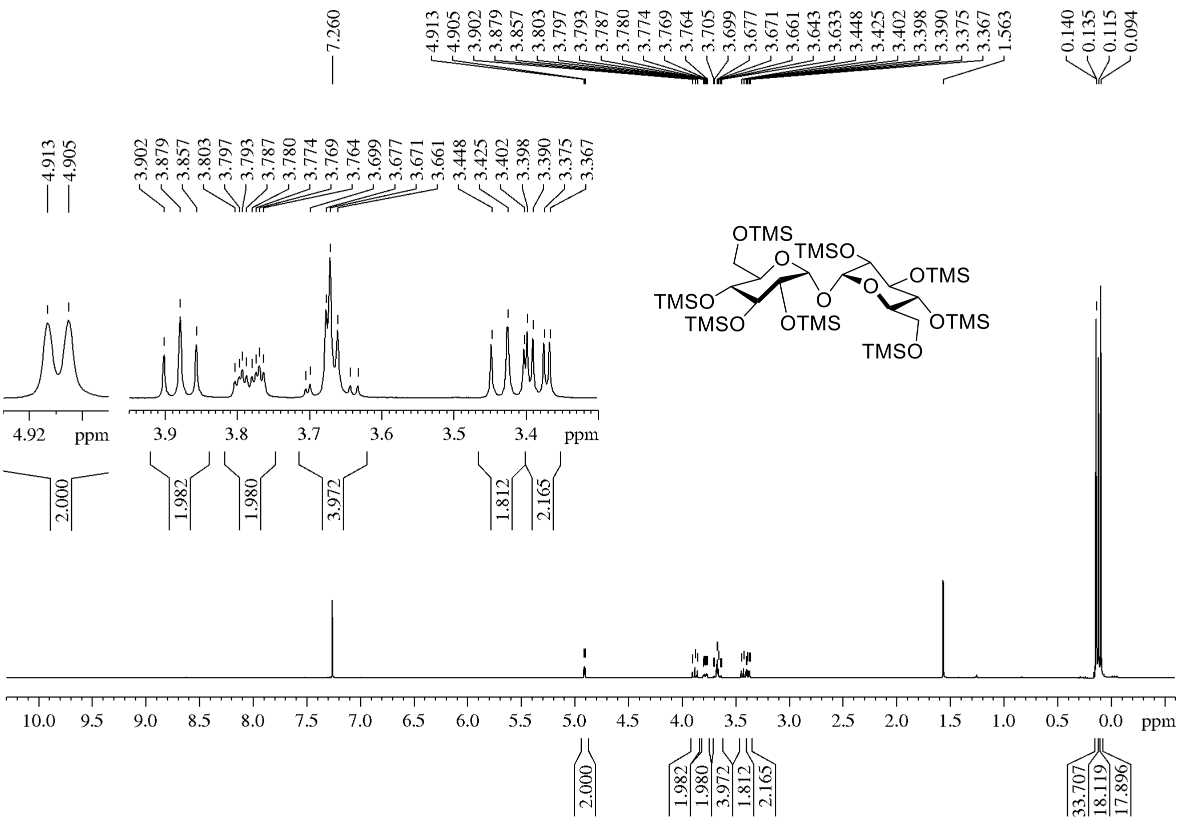

^{1}H NMR of compound **2a** (400 MHz, $CDCl_3$)

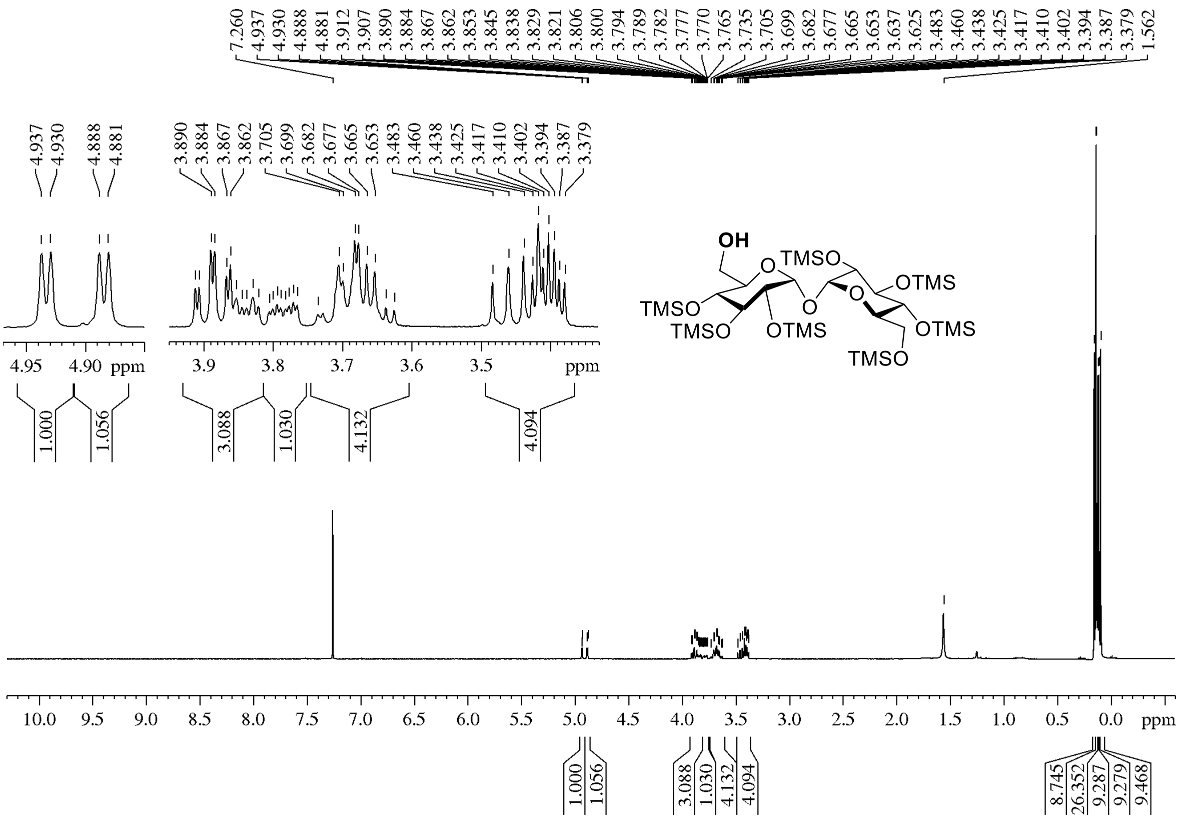

^{1}H NMR of compound **2b** (400 MHz, $CDCl_3$)

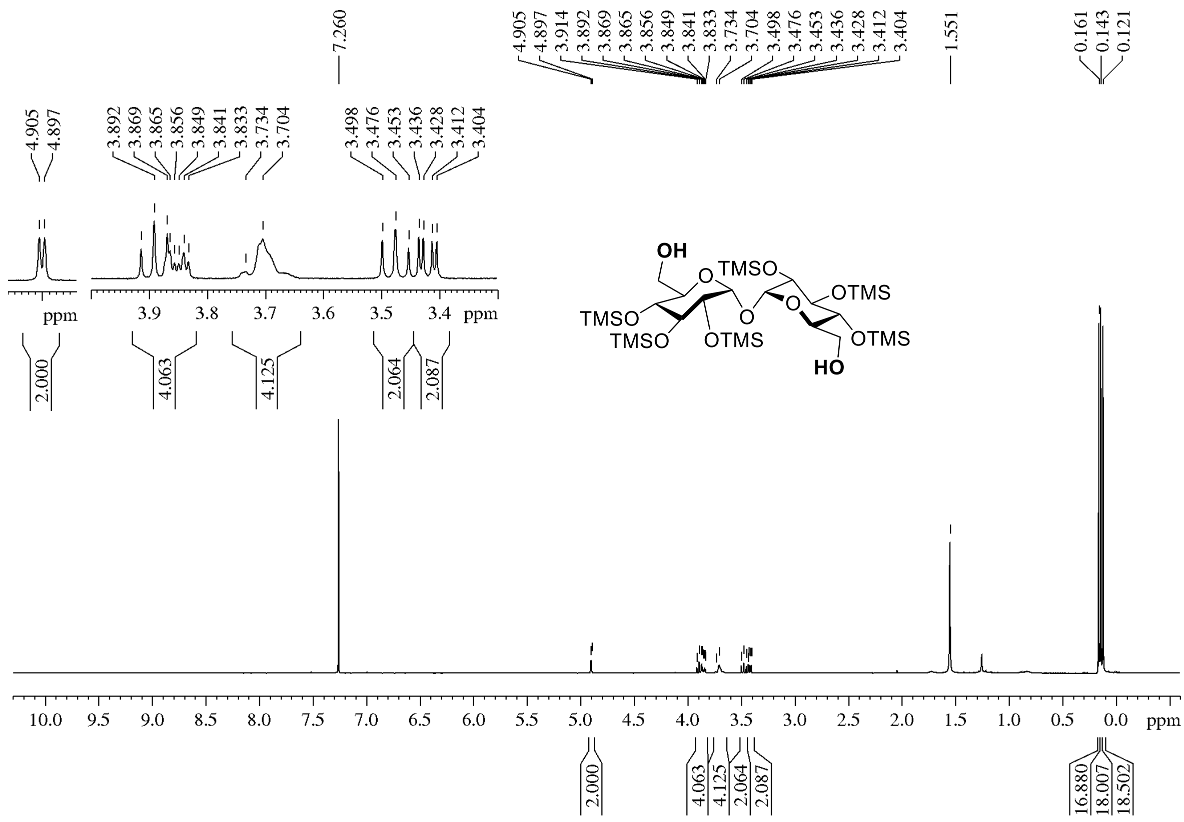

^{1}H NMR of compound **3** (400 MHz, $CDCl_3$)

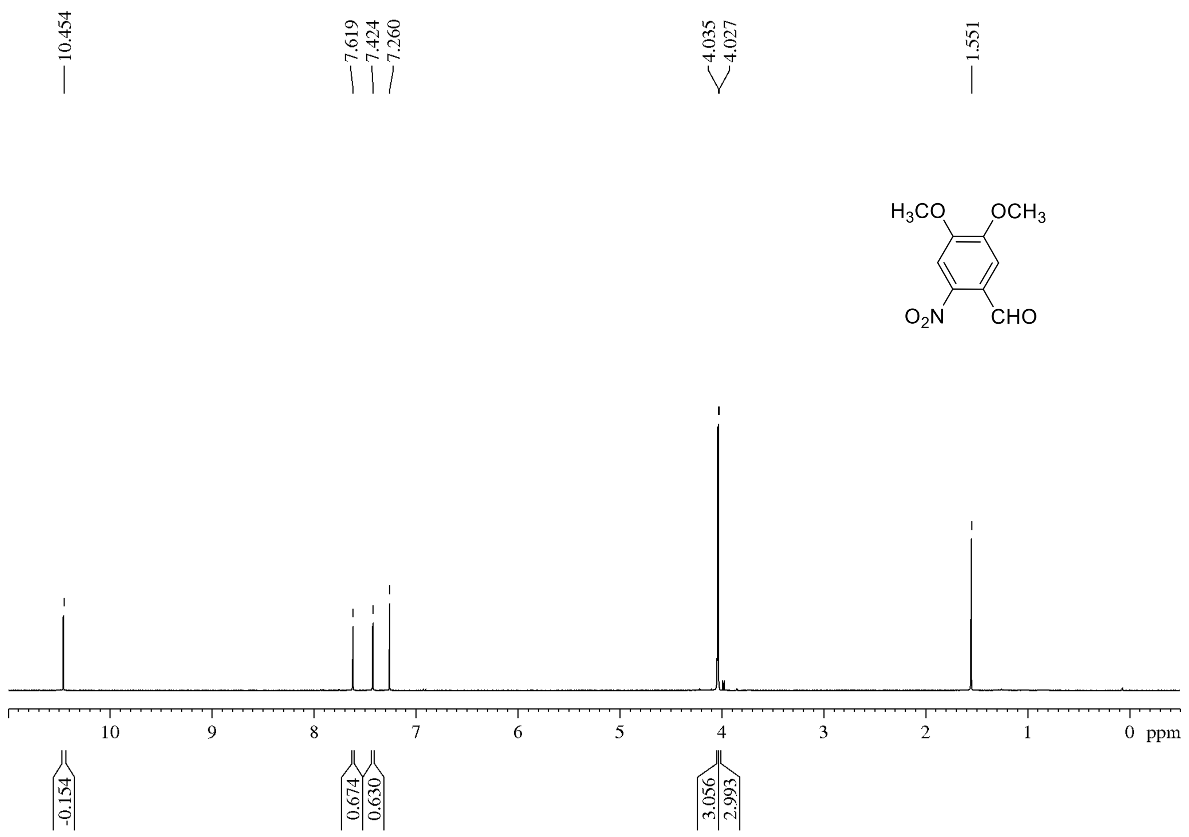

^{1}H NMR of compound **4** (400 MHz, $CDCl_3$)

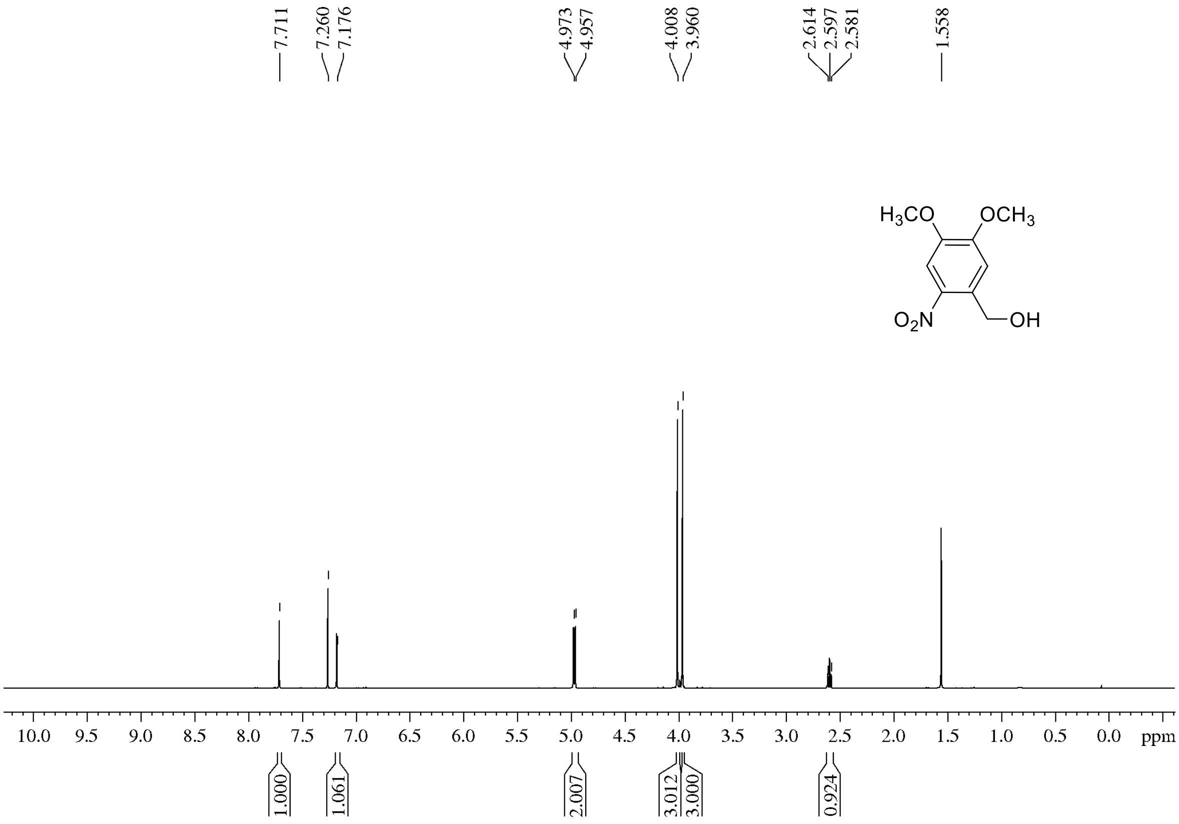

^{1}H NMR of compound **6** (400 MHz, $CDCl_3$)

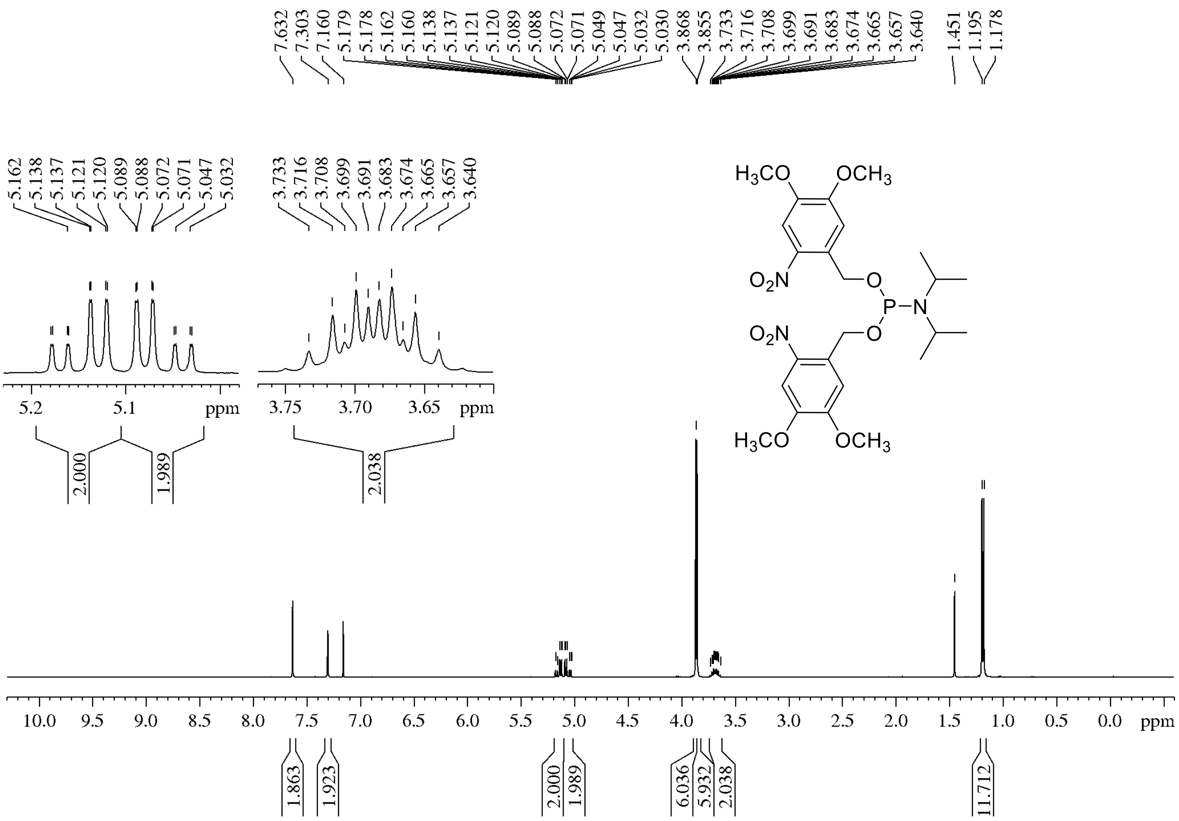

^{13}C NMR of compound **6** (100 MHz, $CDCl_3$)

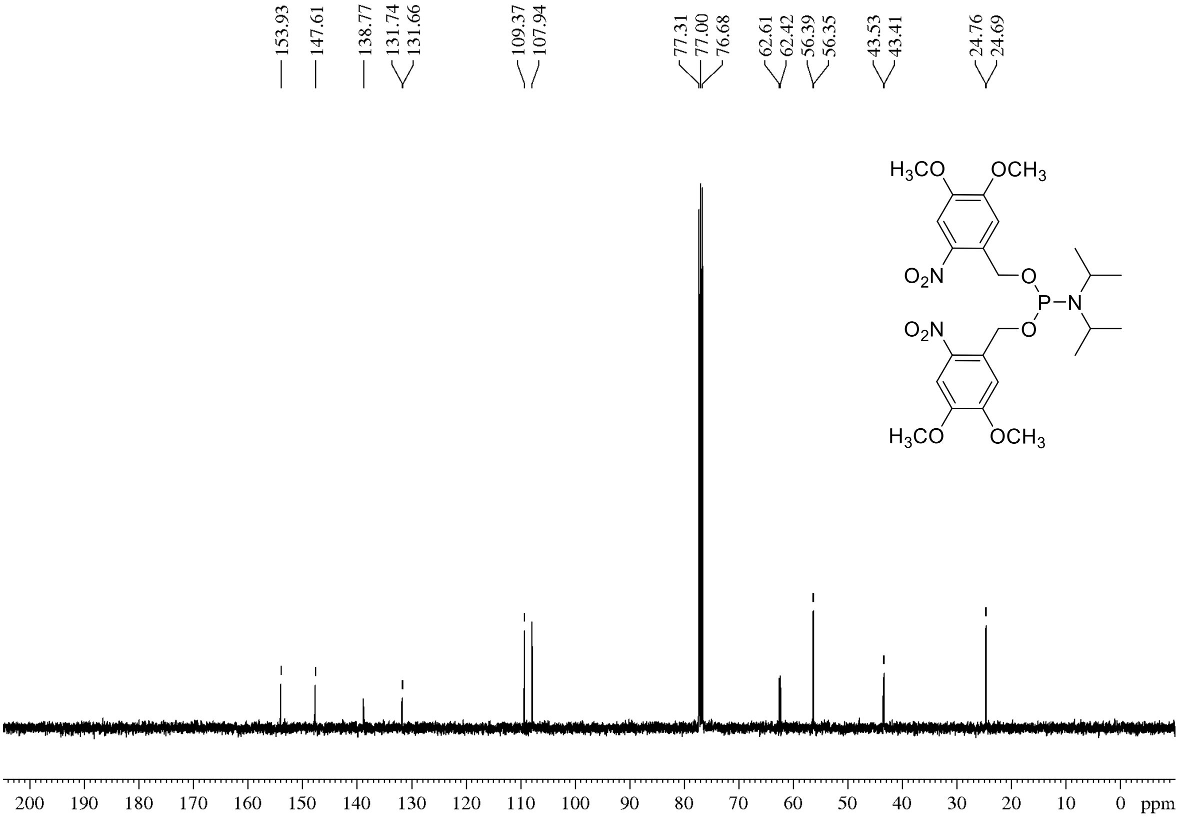

^{31}P NMR of compound **6** (162 MHz, $CDCl_3$)

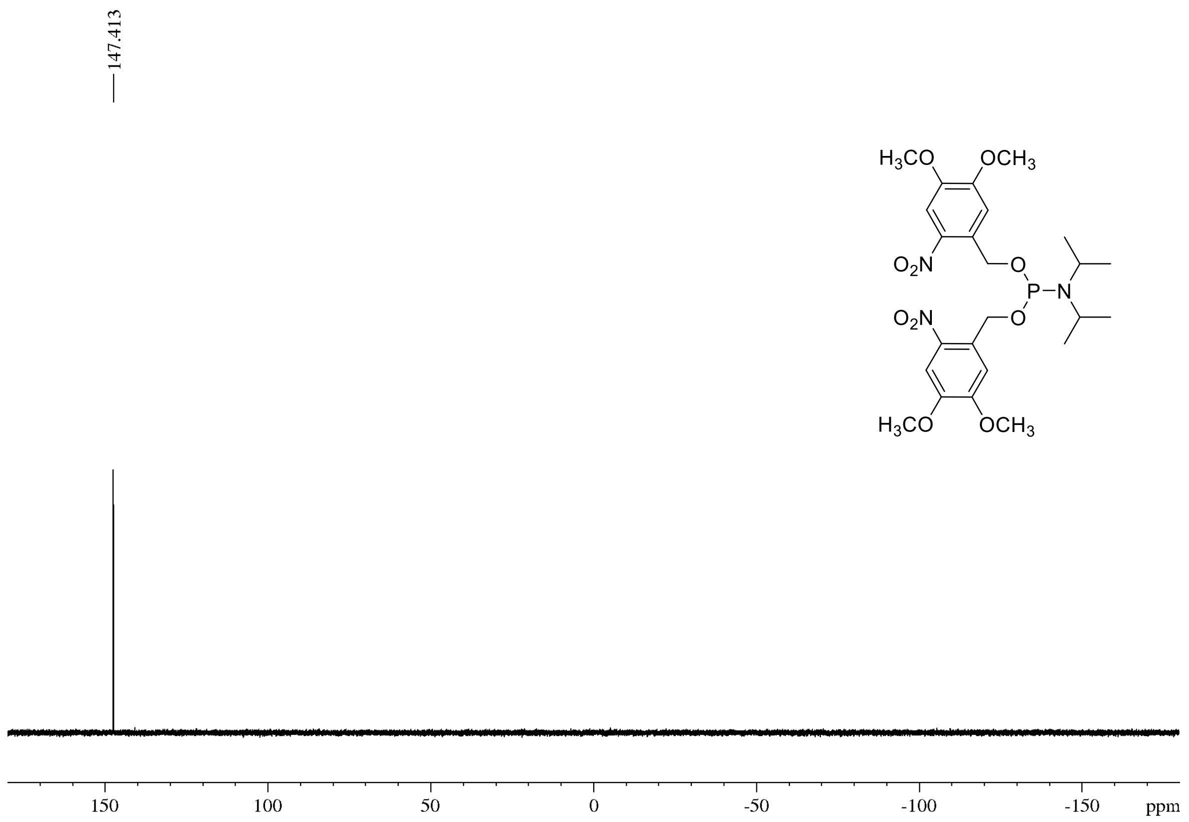

Supplement: Supplementary file 1 — Supplementary Tables 1–6 and Supplementary Chemical Data [file 41587_2025_2611_MOESM1_ESM.pdf]
